# A vagal-to-sympathetic reflex controls lung vascular leak

**DOI:** 10.64898/2026.10.22.761052

**Published:** 2026-10-27

**Authors:** Emily K. Robinson, Emma M. Ibanez, Blanca Santibanez Ocampo, Juan Li, Nupur Ballal, Joelle M. Miller, Kaustubh Shivdikar, Benjamin D. Umans, Sara L. Prescott

**Affiliations:** 1. Department of Biology, MIT, Cambridge, MA, USA; 2. Department of Brain and Cognitive Sciences, MIT, Cambridge, MA, USA; 3. Picower Institute for Learning and Memory, MIT, Cambridge, MA, USA; 4. Biology of Adversity Project, Broad Institute of MIT and Harvard, Cambridge MA, USA; 5. Advanced Micro Devices (AMD), Inc., Boston MA, USA; 6. Department of Otolaryngology-Head and Neck Surgery, Beijing Tsinghua Changgung Hospital, School of Clinical Medicine, Tsinghua University, Beijing, China.

## Abstract

Dynamic regulation of the vascular barrier is required for an effective inflammatory response, allowing delivery of plasma proteins and immune cells to sites of insult. Although normally protective, sustained leak becomes costly in organs like the lung, where swelling stiffens tissue and impairs breathing, yet many upstream regulators remain unidentified. Using a chemogenetic screen of vagal afferent subtypes, we identify a neural reflex that drives lung vascular leak. The reflex originates in an orphan population of neurons expressing *Cysltr2* that innervates alveolar capillary beds and senses both mechanical and inflammatory stimuli, fitting classic definitions of pulmonary “J” receptors, an afferent subtype described anatomically since the 1950s whose function has remained speculative. Activating CYSLTR2 neurons increases lung vascular leak within minutes and causes perivascular edema and respiratory dysfunction, whereas silencing them during inhaled allergen challenge reduces leak and improves lung mechanics. Rather than acting locally through antidromic peptide release, this reflex engages brainstem relays and sympathetic efferents to broadcast its effects throughout lung vasculature. Vagal control of the vascular barrier thus forms an axis of organ defense that can go awry in disease, revealing new ways to regulate organ fluid balance upstream of the endothelium.

## Introduction

The lungs are a uniquely vulnerable interface between the body and the environment, requiring tiers of neurally-coordinated defense. The vagus nerve orchestrates much of this surveillance, densely innervating the airways and other organs to provide moment-by-moment feedback to the brain^1,2^. Vagal neurons evoke protective reflexes including cough and gasp^3,4^, changes in breathing^5–7^, sickness behavior^8^, secretion^9–11^ and immune activation^9–11^. Underlying this functional breadth is a diversity of afferent neural pathways^3,12–16^, yet even in an organ as important as the lungs, many afferent subtypes have no assigned function, raising the possibility that entire classes of vagal reflexes may have eluded description.

One element of lung defense in which the vagal inputs are poorly defined concerns the regulation of the vascular barrier. Regulated increases in endothelial permeability are an important arm of innate immunity across many tissues, facilitating the delivery of immune cells, antibodies, and plasma proteins to sites of insult^17–20^. In the skin, sensory neurons promote vascular leak through the antidromic release of tachykinins, a phenomenon known as neurogenic inflammation^21,22^. Electrical, pharmacological, and vagotomy studies suggest vagal neurons may control vascular leak in the lungs as well^23–25^, but the precise afferent or effector mechanisms involved have not been identified. Furthermore, though some subsets of vagal lung afferents express tachykinins, the sparsity of substance P-positive neurons near sites of barrier disruption^24^ and the failure of tachykinin receptor antagonists in human clinical trials cast doubt on the relevance of a local peptidergic mechanism in the lungs^26^. Vagal neurons may therefore modulate vascular barriers by mechanisms distinct from those employed in the skin.

Although vascular permeability is understood to facilitate the resolution of acute injury, excessive or sustained leak produces edema that compromises respiratory function. The nature of that compromise, and its relative participation in different diseases contexts, depends on which vascular compartment is breached. For example, disruption of the alveolar-capillary barrier causes flooding of the alveolar septa and air spaces that is central to the pathogenesis of pneumonia^27,28^ and acute respiratory distress syndrome (ARDS) ^29,30^, the leading cause of COVID-19 mortality. Alternatively, excessive leak from the bronchial microvasculature causes swelling of the airway wall, exacerbating the airflow restriction and remodeling seen in asthma^31,33^. Finally, leak from post-capillary venules causes peribronchovascular cuffing, fluid buildup in the interstitial zones around large blood vessels, disrupting lung mechanics in conditions like cardiogenic or high-altitude pulmonary edema^32–35^ and vasculitis^32–35^. Altered vagal signaling is documented in many of these conditions^32–35^, although its significance is unclear. Despite decades of work on endothelial barrier regulation, edema cannot yet be treated at its source^36,37^, underscoring a need to identify additional upstream regulators of permeability.

Here we identify a previously uncharacterized vagal sensory population that controls lung vascular leak. These neurons are marked by expression of the cysteinyl leukotriene receptor *Cysltr2*, innervate the alveolar capillary bed, and respond to mechanical and inflammatory stimuli--resembling the pulmonary J (“juxtacapillary”) receptors described by Paintal in the mid-20^th^ century^38,39^. Recurrently activating CYSLTR2 neurons drives fluid accumulation around the airways and vessels and impairs lung mechanics, whereas silencing them during allergic challenge reduces perivascular cuffing and preserves lung function. Unlike leak in the skin, vagally evoked edema requires brainstem relays and sympathetic efferents rather than antidromic release. Together, these findings establish a previously unrecognized vagal-to-sympathetic reflex that has wide implications for both lung health and disease.

## Results

### A chemogenetic screen identifies a vagal subtype that persistently alters lung mechanics

Many respiratory diseases are characterized by tissue changes that persistently alter airway mechanics. While most prior studies of neural influence on lung function have focused on immediate reflex effects, we sought to determine whether persistent or mistuned vagal signaling is sufficient to drive lasting features of airway dysfunction. To assess this possibility, we gained genetic access to lung-innervating vagal neurons using a panel of subtype-restricted *Cre* driver lines. Lung afferents have been identified through both tracing^3,4,15,40^ and lung retrograde labeling studies^13,16^, encompassing at least 10 vagal populations of both known and unknown functions (Fig. 1A, S1A–B). Our *Cre* panel included predicted chemosensory and mechanosensory subtypes, including jugular (*Npy1r-Cre*^3^) and nodose C-fiber (*Vip-Cre*^41^ and *Cysltr2-Cre)* populations, and large-diameter neurons (*Gabra1-Cre*^3,40^, *Calb1-Cre*^3,15^) which innervate pharyngeal and trachealis terminals. Because *Calb1-Cre* exhibits lineage artifacts, we substituted *Kcng4-Cre*, which targets a jugular *Calb1*^+^ subset that recapitulates tracheal chandelier-shaped terminals upon vagal tracing (Fig. S1B). *Vglut2-Cre*, which broadly accesses all vagal afferents^5^, as well as *Piezo2-Cre*, a marker for vagal mechanoreceptors^42^, were included as controls. Together, our panel covers the majority of reported lung chemo- and mechanosensory neurons.

To screen for vagal pathways that shape lung health, we turned to lung function testing. Lung function tests report on the mechanics of breathing, principally resistance (the pressure needed to drive air through the airways, which reflects airway narrowing), compliance (the ease with which the lungs expand for a given pressure, which reflects tissue stiffness), and their temporal fluctuation (the breath-to-breath variability of these values over time that carries independent prognostic information in airway disease^43–45^). Since most commercial systems for mouse lung function testing are incompatible with intravital vagal manipulation, we developed a custom platform that extracts these parameters in real time from a recurrent breath-hold maneuver in anesthetized animals. The design offers two advantages: every breath cycle is resolved rather than sampled intermittently, so we measure fluctuation directly, and the neck remains exposed for vagal access (Fig. 1B; details in supplementary methods). To illustrate the sensitivity of the system, we performed methacholine bronchoprovocation testing, which showed reversible and dose-dependent changes in resistance and compliance (Fig. 1C, top) and recapitulated known sex and age effects (Fig. S1C). A moving window of variation revealed slow, rhythmic oscillations in baseline airway resistance (~0.5 Hz, Fig. 1C, bottom), reminiscent of the airway peristalsis reported in neonatal and juvenile mice and in other species^46–48^. Wave power of these oscillations decreased transiently after each methacholine dose, as expected for a slow, coordinated contraction. To illustrate our platform’s compatibility with vagal manipulations, we performed optogenetic activation of all vagal sensory (*Vglut2-Cre;loxP-ChR2*) or motor (*Chat-Cre;loxP-ChR2*) neurons, which immediately increased airway resistance without altering compliance (Fig. 1D). These effects resolved rapidly after light stimulus cessation. Thus, our platform provides a readout to link genetically-defined neural pathways to both acute and persistent changes in lung mechanics *in vivo*.

These findings confirm that lung mechanical changes are largely reversible following transient vagal activation. We next performed a chemogenetic screen across our *Cre* line panel to constitutively activate vagal pathways for several days, mimicking the recurrent activation often seen with naturalistic stimuli, then incorporated a washout period (Fig. 1E) to isolate effects that persist beyond neuronal activation. This strategy was designed to remove acute reflex components and instead unmask previously-hidden slower effects with potential disease relevance, such as reflex circuit plasticity, lung structural remodeling, immune sequelae or other lasting tissue changes. For each *Cre* line, we delivered a *Cre*-dependent excitatory DREADD^49^ (*AAV-Gαq-DREADD)* to the vagal ganglia by AAV injection. After 3 weeks of recovery, we administered the synthetic ligand compound 21 (C21)^50^ twice daily for 3 days by intraperitoneal injection followed by a 2-day washout before terminal lung function testing. Activation of all vagal sensory neurons (*Vglut2-Cre*) led to an increase in airway resistance, demonstrating that repeated vagal activation drives lasting mechanical changes in the absence of lung injury or a primed allergic response (Fig. 1E, left). Activation of PIEZO2-, NPY1R-, KCNG4-, VIP-, or GABRA1-defined vagal subpopulations produced no lasting effects. Strikingly, activation of CYSLTR2 neurons produced a sustained elevation in resistance, decrease in compliance, and heightened airway lability (Fig. 1E, right). Because CYSLTR2 neurons are a population of unknown function, we sought to further characterize their role in lung physiology.

### CYSLTR2 neurons are alveolar “juxtacapillary” (J) receptors

We first examined CYSLTR2 neuron identity. Sensory neurons have historically been classified in part by their end terminal location and morphology, so we mapped CYSLTR2 neurons to their peripheral targets using genetically-guided viral tracing. CYSLTR2 neuron innervation outside the lung was sparse, limited to the heart and gut mucosa and consistent with *Cre* expression in other rare sensory subtypes (Fig. S2A). Because the *Cysltr2-Cre* line had not previously been validated in vagal neurons, and to assess potential lineage effects, we also performed two-color RNA *in situ* hybridization against *Cysltr2* and *ChR2* transcripts in the vagal ganglia of *Cysltr2-Cre;loxP-ChR2* mice. Few cells expressed *ChR2* without detectable *Cysltr2* expression (~8% of all *Chr2*-expressing cells, Fig. S2C,D), indicating limited off-target effects.

Within the lung, the majority of traced CYSLTR2 fibers extended along major bronchioles before diverting into the alveolar compartment (Fig. 2A). Individual fibers then branched and encircled one or multiple alveoli before terminating within a CD31+ capillary bed (Fig. 2B, Movies 1 and 2). At higher magnification, interseptal capillary terminals took two forms: flattened elaborate arbors (“tree branch”), or small beaded (“rosebud”) endings. Both intercalated near capillary junctions, with occasional terminals also near neuroepithelial bodies (Fig. S2B). This terminal position within the alveolar wall is the defining anatomical criterion of pulmonary J (“juxtacapillary”) receptors.

Classically, pulmonary J receptors were defined as slowly-conducting neurons with dual sensitivity to specific chemicals (serotonin) and mechanical stimuli (raised capillary pressure, lung hyperinflation)^51^. *Cysltr2* marks a population of C-fiber lung afferents of nodose origin (*Phox2b*^+^, *Nefh*-low, *Scn10a*^+^, *Htr3a*^+^, *Piezo1*^+^) that potentially fits this profile. To ask if CYSLTR2 neurons share these sensory properties, we tested the responses of CYSLTR2 neurons to classic pulmonary J receptor stimuli *in vitro*. We dissociated vagal neurons from *Snap25-GCaMP6s;Cysltr2-Cre;loxP-tdTomato* mice and recorded calcium responses in CYSLTR2^+^ and CYSLTR2^−^ neurons in parallel during *ex vivo* perfusion (Fig. 2D, 2E). CYSLTR2^+^ neurons had enriched responses to serotonin, the prototypical J receptor agonist, compared to CYSLTR2^−^ neurons (59.5% versus 41%, respectively). In contrast, responses to bradykinin and histamine, which act on other lung C-fiber classes but spare J receptors, were depleted. Given the expression of *Cysltr2*, we also tested their responsiveness to cysteinyl leukotrienes C4 and D4^52,53^. These did not elicit detectable calcium responses in any neurons tested, suggesting a modulatory rather than directly excitatory function (Fig. S2E).

Another criterion for J receptors is their sensitivity to mechanical cues. Interestingly, while lunginnervating CYSLTR2 neurons do not express mechanosensitive ion channel *Piezo2* (a marker for most visceral mechanoreceptors), they express high levels of *Piezo1*. We therefore tested calcium responses to the *Piezo1* agonist Yoda1. We observed Yoda1-induced activation of a subset of CYSLTR2 neurons, consistent with the *Cysltr2-Cre* expression encompassing the *Piezo1*^+^ lung afferent subtype; Yoda1 responses were observed in CYSLTR2^−^ neurons as well, as expected given the presence of *Piezo2*^+^*Piezo1*^+^*Cysltr2*^−^ baroreceptors that innervate the aortic arch^51^. Thus, CYSLTR2 neurons fit the anatomical and sensory criteria of pulmonary J receptors, providing a genetic handle to interrogate their function.

### CYSLTR2 neurons cause tissue congestion by inducing vascular leak

We therefore sought to determine how their persistent activation impairs lung function. We first histologically examined the lungs of the chemogenetically-activated *Cysltr2-Cre* mice to look for structural clues. Their lungs showed no evidence of smooth muscle hypertrophy, fibrosis, goblet cell hyperplasia, or excessive immune infiltration (Fig. S3A). The only structural correlate was perivascular cuffing, a sign of fluid overload in the interstitial spaces surrounding the large blood vessels that often precedes more severe alveolar edema (Fig. 3A, 3B). Interstitial fluid accumulation around the bronchovascular bundle can disrupt lung function by compressing airway walls and mechanically uncoupling them from surrounding parenchyma--findings that could explain both the increased resistance and decreased compliance seen in our lung function tests^54^. Because cuffing is typically considered a leading rather than lagging sign of abnormal fluid homeostasis, we next turned to more acute readouts of edema. We therefore applied vagal optogenetics and measured the post-mortem gravimetric lung wet-to-dry (W/D) ratio, a standard metric of edema^55,56^. Even just a brief 10-minute stimulation (20Hz, 5ms) of CYSLTR2 neurons raised the wet-to-dry (W/D) ratio by ~45% compared to sham-stimulated animals, exceeding the effect of intratracheal histamine over the same interval (~23% W/D ratio increase) (Fig. 3C). This rapid rise indicates extravascular fluid accumulation.

Fluid accumulation in the lungs can have various causes. Since peribronchovascular cuffing is frequently seen in cardiogenic (heart-related) pulmonary edema, we asked if this fluid buildup was secondary to impaired cardiac function. Unlike VGLUT2 neuron stimulation, which evoked immediate tachypnea and bradycardia, transient (10-second) activation of CYSLTR2 neurons had no immediate effects on heart rate or breathing, suggesting that the edema is non-cardiogenic and precedes later respiratory dysfunction (Fig S3B, S3C). Another possibility is that CYSLTR2 neurons evoke edema by increasing endothelial permeability. We therefore assessed vascular barrier integrity using an Evans blue assay, a blue dye that binds albumin and allows direct quantification of plasma leak. A sustained 10-minute optogenetic activation of either VGLUT2 or CYSLTR2 neurons induced profound endothelial barrier disruption. After neuron activation, lungs were visibly bluer and had greater tissue congestion than control-stimulated lungs, similar to the lungs of histamine-treated mice (Fig. 3D), indicating accumulation of plasma albumin. Lung dye extraction confirmed ~54% increase in extravasation following CYSLTR2 neuron activation compared to sham controls (Fig. 3E). Thus, CYSLTR2 neuron activation rapidly disrupts the vascular barrier, causing peribronchovascular edema that impairs lung function.

### CYSLTR2 neurons are engaged during an inhaled ovalbumin challenge

Having found that activating CYSLTR2 neurons is sufficient to drive lung peribronchovascular leak, we asked whether these neurons participate in tissue congestion caused by an inhaled challenge. Cuffing (sometimes described as a ‘donut sign’) is a common radiologic sign in airway allergy^57,58^. Therefore, we turned to an inhaled ovalbumin (OVA) model for its simplicity, high degree of vascular involvement^57,58^, and compatibility with genetic manipulation. Mice were sensitized with OVA in aluminum hydroxide (alum) on days 0 and 7, then challenged with inhaled OVA on three consecutive days; controls received alum alone followed by inhaled saline (PBS). By day 20, OVA-challenged animals exhibited increased serum OVA-specific IgE levels, immune infiltration, and tissue remodeling, confirming an allergic response (Figs. 4A, S5A, S5B, S5C, S5F). As expected, the lungs of OVA-treated mice showed pronounced perivascular cuffing, with many vessels surrounded by an edematous zone with immune infiltration (Fig. 4A, S5E). This was associated with lung function impairment, marked by increased resistance and reduced compliance—collectively a stiffer, flow-limited phenotype (Fig. 4B, S5D). Airway hyperresponsiveness was not observed, as commonly reported for allergen models in the C57BL/6 background^59^.

To ask whether CYSLTR2 neurons participate in OVA-induced vascular edema, we turned to single-cell RNA sequencing. Since activated vagal neurons are not effectively labeled by c-FOS, we reasoned alternatively we could use changes in gene expression to predict functionally-engaged subtypes. We harvested vagal ganglia from mice subjected to OVA or PBS challenge, collected at the peak of airway inflammation (day 20), or 8 weeks following the last OVA or PBS challenge (Fig. 4C) to assess signs of long-lasting transcriptional memory. In total we captured ~323K cells across the four conditions, including vagal neurons, sympathetic neurons from the nearby superior cervical ganglion, satellite glia, Schwann cells, immune cells, endothelial cells, and fibroblasts (Fig. S4A; n=5 male mice per replicate, four replicates per condition). Post-filtering we recovered 129,810 sensory neurons which clustered into 36 vagal and glossopharyngeal populations, which we enumerated as J1-10 and N1-26, for “jugular” and “nodose/petrosal” (Figure 4D). Each subtype mapped cleanly to previously published vagal/glossopharyngeal atlas clusters (Fig S4B). All subtypes were recovered in similar proportions across conditions, with no evidence of cluster depletion or transdifferentiation following OVA treatment, indicating that cell-type identity was preserved (Fig. 4D, S4C). While statistically-significant gene expression changes were observed in a subset of clusters during the peak of airway inflammation (day 20), nearly all gene expression changes reverted by 8 weeks post-recovery despite lasting lung function deficits (Fig. S4D, S4E). This demonstrates that ovalbumin-induced airway challenge elicits robust and reversible transcriptional responses in a subset of vagal sensory neurons.

To prioritize vagal subtypes with the strongest responses to inhaled OVA, we next ranked clusters by (i) per-cluster counts of differentially expressed genes in the acute timepoint, and (ii) scDist^60^, a mixed-effects framework for quantifying transcriptional shifts that accounts for inter-animal variability and differences in cluster size. Each method independently converged on two nodose populations with the largest transcriptional responses—N10 and N12 (Fig. 4G). Both subtypes preferentially innervate the lungs in retrograde labeling studies^14,17^, supporting the biological relevance of our analysis. The strongest responder, N10, corresponds to a lung afferent subtype marked by *Vip* that was recently shown to drive allergen-induced airway hyperresponsiveness^41^. The second class, N12, corresponds to the lung afferent subtype targeted by *Cysltr2-Cre*, confirming their functional engagement during OVA-induced airway inflammation. The major markers and receptor profiles of both populations remained largely unchanged by the OVA challenge; however, gene set enrichment analysis (GSEA) predicted changes in genes related to neuronal plasticity, TNFα–NFκB, and JAK–STAT3 signaling pathways (Fig. 4F, Table S3). Both N10 and N12 neurons strongly express the TNFα receptor *Tnfrsf1a* and Janus kinase (JAK) family machinery *Jak1/Stat3/Stat6* (Fig S4F), consistent with the known roles of TNFα and JAK-STAT signaling in airway allergy and neuronal sensitization^1,61^, providing a cell-intrinsic basis for their favored engagement.

The observation that only 2 out of 36 vagal populations respond to OVA challenge supports the labeled-line model of viscerosensory processing, and motivated closer comparison of their molecular identities (Fig. 4E). Both N10 and N12 are placode-derived (*Phox2b*^+^) and express a broad repertoire of receptors for inflammatory mediators (*Tnfrsf1a*, *Il13ra1*, *Nod1*, *Ednra*, *S1pr3*, *Ptger2/4*). They diverge however in core identity. N10 neurons are prototypical C-fibers, marked by TRP channels (*Trpv1/Trpa1*), neuropeptides (*Calca/Calcb, Vip*), and sodium channels (*Scn10a*, *Scn11a*). N12 neurons, by contrast, lack TRP channels and sensory neuropeptides (*Tac1/2/3*, *Calca/Calcb*, *Vip*) and instead express a combined mechano-/chemosensory module (*Adgrl2*, *P2rx3*, *P2ry1*, *Htr3a/b*, *Scn7a*, *Scn10a*), including *Piezo1* but not *Piezo2*, as described above (Fig. S4G). The observation that N12 neurons lack *Trpv1* expression was notable given the widespread use of capsaicin to identify vagal C-fibers, despite reports of capsaicin-insensitive C-fibers *in vivo*^62^. We therefore tested capsaicin responsiveness of CYSLTR2 neurons using *ex vivo* calcium imaging and found that all Yoda1-responsive *tdTomato*-marked neurons were universally capsaicin-insensitive (Fig S4H, S4I). Together, this identifies two distinct pathways that are preferentially engaged during allergic airway inflammation, including one comprised of a *Cysltr2*^+^*Piezo1*^+^*Trpv1*^−^ neuronal subtype.

### Silencing CYSLTR2 neurons mitigates edema and restores lung function during OVA challenge

Given that more than one vagal pathway was activated by OVA, we asked whether silencing CYSLTR2 neurons alone mitigates OVA-induced pathology. We injected the vagal ganglia of *Cysltr2-Cre* mice with virus carrying a *Cre*-dependent inhibitory DREADD (*AAV-Gi-DREADD-mCherry).* We inhibited CYSLTR2 neurons with C21 (twice daily, i.p.) throughout the OVA aerosol challenge, after immune memory has already formed (Fig 4H). CYSLTR2-silenced mice had similar levels of OVA-specific IgE serum levels, goblet cell hyperplasia, and total immune cell burden, indicating that their allergic response was not impaired (Fig. S5A–C). However, compared to OVA-treated animals injected with an *AAV-mCherry* control virus, the lung function of CYSLTR2-silenced mice was significantly improved, showing reduced airway resistance, instability, and elastance (Fig. 4J, S5D). Evaluation of lung histopathology also showed significantly reduced cuffing, particularly around large-diameter vessels, but unchanged airway wall and alveolar interseptal thickness, suggesting a regionalized effect on post-capillary venule permeability (Fig 4I, 4K, S5E–F). Silencing of CYSLTR2 neurons therefore prevents tissue congestion.

### The vagal vascular leak reflex is mediated through central and not peripheral pathways

Neurogenic vascular leak can be mediated through either local (antidromic) or central mechanisms. In the skin, somatosensory neurons drive leak by releasing tachykinins onto nearby endothelial and immune cells, leading to the characteristic ‘wheal-and-flare’ of allergic skin reactions. However, CYSLTR2 neurons lack *Tac1/2/4* (Fig. S4G) and are located at a distance from the site of fluid accumulation, arguing against a local antidromic mechanism. Additionally, substance P-positive neurons are sparse in humans, and not found the vicinity of leaky vessels^24,26^, casting doubt on the importance of this pathway in the lung. We therefore considered a model in which vagally-evoked leak involves central reflex circuits.

To distinguish peripheral (antidromic) from central (orthodromic) mediation, we combined targeted chemogenetic activation with nerve cuts. *Cysltr2-Cre* mice were injected unilaterally with an excitatory DREADD (*AAV-flex-Gq-DREADD-mCherry*), and one vagal trunk was transected either ipsilaterally below the vagal ganglion, eliminating peripheral axons while preserving central connections, or contralaterally, preserving both (Fig, 5A). We did not cut above the ganglion because the vagal rootlets are surgically inaccessible inside the murine skull. After 2-week recovery, we unilaterally activated CYSLTR2 neurons with intraperitoneal C21 and measured lung function. Unilateral activation without a nerve cut, or with a contralateral nerve cut, showed lung function impairment comparable to uncut bilateral CYSLTR2 activation (Fig. 5A,S6A). Ipsilateral nerve cuts also did not abolish the DREADD-induced changes in resistance and compliance, indicating that antidromic signaling was not required for the effect (Fig. 5A). Thus, the effects of CYSLTR2 neurons on lung function are mediated at least partly through central relays rather than by direct action of sensory neurons on lung tissue.

Given that the reflex requires brainstem relay, we characterized the central projection patterns of CYSLTR2 neurons. Vagal ganglia of *Cysltr2-Cre* mice were injected with both a *Cre*-dependent *AAV-flex-tdTomato* and *Cre*-independent *AAV-eGFP* to visualize *Cre*-expressing neurons in the context of all types of vagal sensory fibers. While GFP^+^ fibers spanned the nucleus tractus solitarius (NTS), area postrema (AP), and paratrigeminal nucleus (Pa5), CYSLTR2 neurons arborized in an anatomically restricted manner, with highest labeling in the dorsomedial NTS, ipsilateral area postrema, and commissural regions caudal to the obex--regions associated with pulmonary C-fiber input^63,64^ (Fig. 5B, S6B). To exclude signals coming from fibers of passage, we combined chemogenetic activation of CYSLTR2 neurons with c-FOS mapping. Activation increased c-FOS within 90 minutes in the dorsomedial NTS and area postrema, with strongest labeling near −7.43 mm from bregma. Because c-FOS mapping marks downstream circuits as well, we noted increased c-FOS in both the dorsal motor nucleus of the vagus (DMX) and rostral ventrolateral medulla (RVLM), major parasympathetic and sympathetic relay nuclei, respectively (Fig. 5C-D, S6C). This delineates a central pathway through which CYSLTR2 neurons access both parasympathetic and sympathetic outflow to the airways.

### Sympathetic neurons mediate the efferent arm of the vagal vascular leak reflex

We next asked whether the efferent arm of the reflex is sympathetic or parasympathetic. Pulmonary sympathetic fibers outnumber parasympathetic fibers near large pulmonary vessels (Fig S6D), positioning them as the likely effectors of CYSLTR2 neuron-mediated leak. We therefore optogenetically activated the vagosympathetic trunk in *Dbh-Cre;loxP-ChR2* and *Chat-Cre;loxP-ChR2* mice, which mark sympathetic and parasympathetic motor neurons respectively, and measured Evans blue extravasation (Fig. 5E). CHAT stimulation did not raise pulmonary dye beyond sham stimulation. By contrast, DBH stimulation disrupted the endothelial barrier to a degree comparable to CYSLTR2 activation, while sparing skin and abdominal organs innervated by more posterior sympathetic ganglia. Chemical sympathectomy blocked the Evans blue extravasation evoked by CYSLTR2 activation entirely (Fig. 5F). Because CYSLTR2 neurons also innervate other viscera, we asked whether this reflex is confined to the lung. Activation increased Evans blue extravasation in the heart and gut mucosa, while sparing the liver and skin (Fig. 5E–F). Direct stimulation of the vagosympathetic trunk engages cervical and upper thoracic outflow alone, whereas afferent activation recruits medullary relays with access to more posterior sympathetic ganglia, accounting for the difference in the organs affected. Vascular leak in diverse organs is therefore under direct sympathetic control, completing a reflex arc that runs from juxtacapillary afferents through medullary relays to sympathetic effectors.

## Discussion

Airway defense depends on the interplay of many vagal afferent classes, including dedicated pathways that respond to overinflation^42^, airway closure^4^, aspiration^3^, and various irritants^6,40,41^. Here, we describe a reflex initiated by an orphan population of vagal neurons that controls lung vascular leak. Recurrently activating these neurons causes fluid accumulation around the large vessels and bronchi, impairing lung mechanics independent of other airway insults. Single-cell profiling and functional silencing experiments confirm that these neurons are preferentially engaged during allergic inflammation and required for allergen-induced perivascular edema. These neurons act through brainstem relays that engage the sympathetic nerves of the upper chest, coordinating fluid balance across multiple organs, in contrast to the local mechanisms used by edema-promoting nociceptors in the skin. Together, we propose that CYSLTR2 neurons function as vascular state sensors, detecting mechanical and inflammatory compromise of the alveolar capillary bed and directing post-capillary extravasation, perhaps to support local repair and host defense. Under this model, the reflex is useful when engaged briefly and harmful when sustained, since the same extravasation that provisions the tissue also produces cuffing that eventually stiffens the lungs and disrupts breathing.

The subtype-resolved mapping of vagal afferents afforded by modern genetic tools is helping to clarify long-standing questions about lung C-fibers. For example, Paintal originally defined J receptors by their response to vascular mechanical stimuli, whereas later work defined them instead by their response to capsaicin. The two definitions are difficult to reconcile, because capsaicin-responsive neurons react only weakly to raised capillary pressure and lung inflation^65,66^. Furthermore, Paintal proposed that J receptor cause dyspnea (breathlessness) and exercise termination, yet human volunteers injected intravenously with capsaicin do not report dyspnea^73^. Our work may help explain these discrepant observations by showing that there are at least two subtypes of alveolar endings, that together account for features collectively ascribed to J receptors. Of the alveolar subtypes, N10 neurons (marked by *Vip*) respond to capsaicin have been shown to initiate airway hyperresponsiveness^45^, whereas N12 neurons (marked by *Cysltr2*) respond to mechanical stimuli but not TRP channel agonists and evoke leak from large pulmonary vessels. Other C fiber classes exist as well – for example, the airway wall mucosa is innervated by jugular nociceptors (marked by *Npy1r*, *Trpv1* and *Tac1* expression*)*^74^, which drive tachypnea and urge-to-cough^3,75^, and may also initiate vascular leak from the bronchial microvasculature. Natural insults likely engage more than one pathway at once, as we see for N10 and N12 neurons during OVA challenge, and the relative weights of pathway activation may account for different symptomatic outcomes seen across individuals or exposure types. It also leads to the tempting hypothesis that targeting one pathway could resolve some reflex outcomes while sparing others needed for eupnic breathing.

One implication of this work is that neural dysregulation could drive airway pathology rather than merely respond to it. Leakage of plasma from airway vessels is a recognized feature of many airway disorders, but has largely been viewed as a downstream consequence of inflammatory mediators acting directly on the endothelium^70,71^. Our results place both vagal and sympathetic neurons upstream of that process. Sympathetic control of pulmonary vascular leak is already recognized in the context of specific pathologies, like neurogenic and high-altitude pulmonary edema, but the evolutionary purpose of this adrenergic sensitivity was unclear. In those cases, sympathetic neurons are thought to evoke edema through changes in hydrostatic pressure, redistribution of blood volume, cardiac effects, reduced clearance, or direct effects on endothelial permeability^72^. In aggregate, we suggest that the sympathetic nervous system is not simply a source of pathological catecholamine excess, but an effector through which sensory information about the state of the lung can be translated into coordinated changes in organ fluid balance.

Our work also raises the possibility that neurotherapeutics could provide targeted benefit for conditions associated with excessive peribronchovascular leak. Cysteinyl leukotrienes themselves have well known roles in airway allergic disease, with CYSLTR1 antagonists in clinical use and CYSLTR2 variants associated with asthma and aspirin-exacerbated respiratory disease^73–75^. Our results suggest that these and other inflammatory mediators may also contribute to disease by engaging neural circuits that drive lung pathology, representing a new direction for clinical investigation. From these combined results, we conclude that CYSLTR2 vagal neurons constitute a neural axis controlling lung fluid balance in both health and disease.

## Supplementary Material

Supplement 1

Supplement 2

Supplement 3

4

## Figures and Tables

**Fig. 1.**
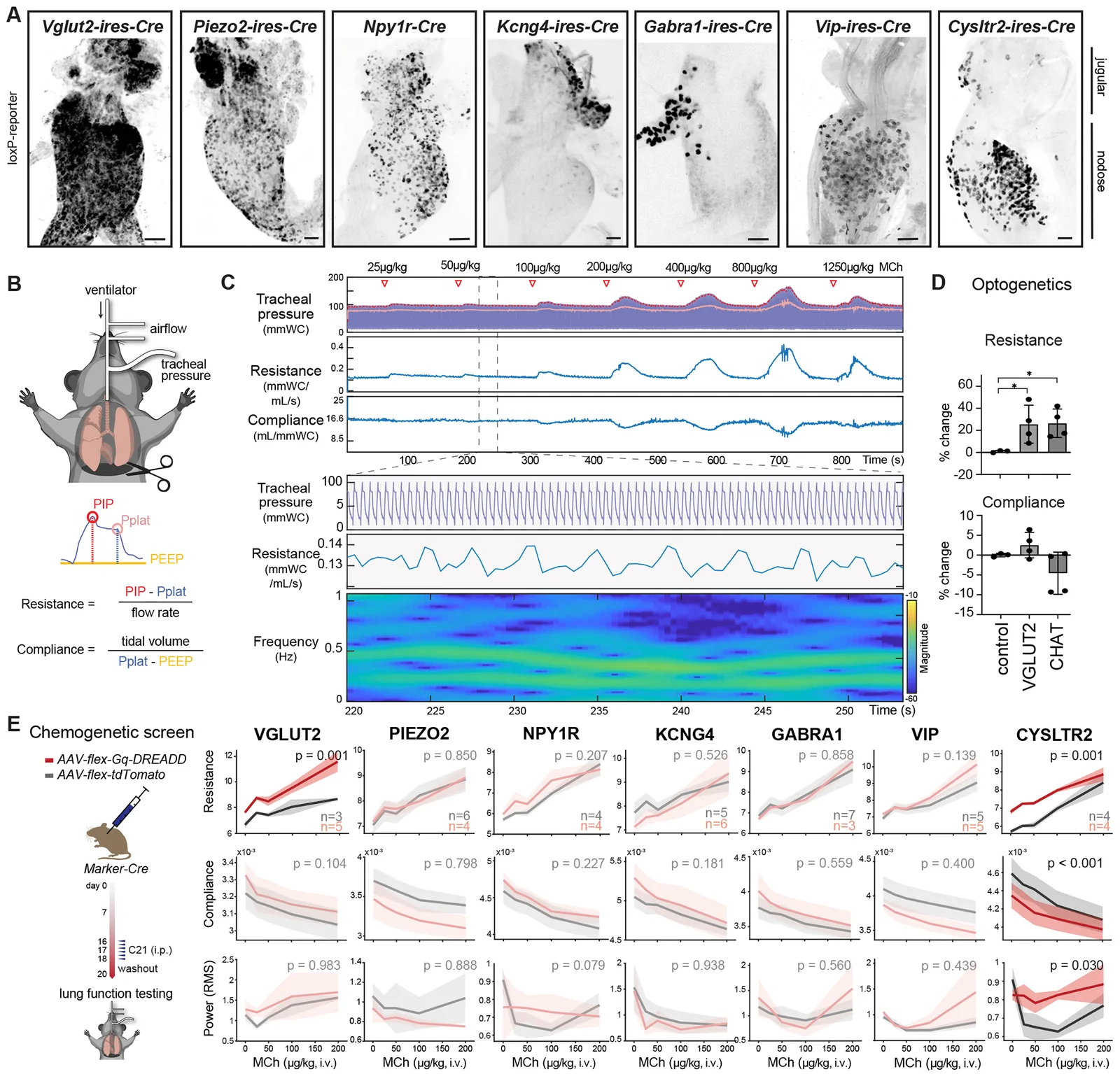
A chemogenetic screen identifies a lung afferent subtype that persistently alters airway mechanics. **(A)** Vagal ganglia from mice carrying *Cre* alleles and *Cre*-dependent reporters marking seven lung-innervating vagal sensory populations (*loxP-ChR2-eYFP* for *Vglut2-ires-Cre*, *Gabra1-ires-Cre, Vip-ires-Cre* and *Cysltr2-ires-Cre*; *loxP-DTR* for *Piezo2-ires-Cre*, *Kcng4-Cre*, and *Npy1r-Cre*). Scale bars, 100 μm. **(B)** Lung function testing paradigm and derived parameters. **(C)** Top: representative tracheal pressure trace (mmWC; 10 mmWC = 1 cmH_2_O) with peak inspiratory (red) and plateau (pink) pressures marked during methacholine (MCh) challenge in a wildtype animal, above the derived resistance (mmWC/mL/s) and compliance (mL/mmWC) measurements. Bottom: expanded view of breath-to-breath resistance oscillations and a spectrogram of detrended resistance showing spectral power (dB) versus frequency (Hz) and time. Color axis represents the magnitude in dB relative to 1 mmWC·min/mL. **(D)** Change in resistance and compliance during optogenetic stimulation of the vagosympathetic trunk in mice expressing *ChR2* from the indicated *Cre* drivers. n = 3–4 mice per line; two-tailed unpaired t test; *P < 0.05, **P < 0.01. **(E)** Left: design of the DREADD-based chemogenetic screen. Right: resistance (AUC), compliance (median), and wave power (AUC of RMS spectral power) over the 60 s after each MCh dose (i.v.; 25, 50, 100, 200 μg/kg) in mice injected with *AAV-Flex-tdTomato* (control, gray) or *AAV-hSyn-DIO-hM3D(Gq)-mCherry* (red). See supplementary materials for additional information on lung function metrics. P-values were calculated using an omnibus model with dose as a categorical factor, group × dose interaction reported; Data are means ± SEM throughout.

**Fig. 2.**
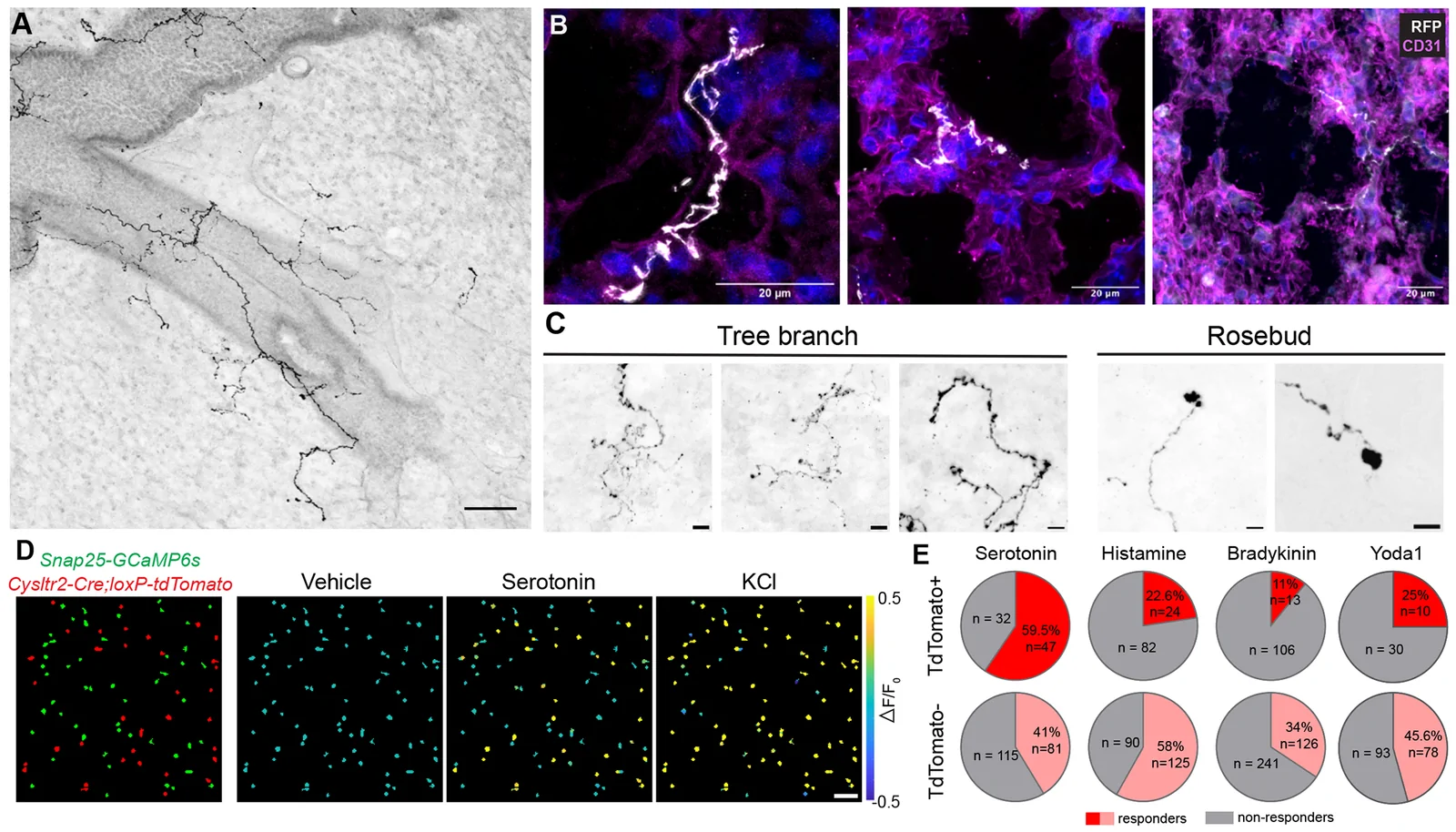
CYSLTR2 neurons are pulmonary J receptors. **(A)** Labeled terminals in a 300-μm lung slice from *Cysltr2-ires-Cre* mice injected in the nodose/jugular/petrosal (NJP) ganglia with *AAV-Flex-tdTomato*. Scale bar, 100 μm. (B) *Cysltr2*^+^ terminals (white) adjacent to alveolar structures labeled by CD31 (magenta), with DAPI nuclear counterstain in blue. Scale bar, 20 μm. **(C)** Example morphologies of *Cysltr2^+^* terminals. Scale bars, 10 μm. **(D)** Calcium imaging of dissociated vagal neurons from *Cysltr2-Cre;loxP-tdTomato;Snap25-GCaMP6s* mice during sequential stimulus perfusion. Left: Snapshot showing all vagal neurons expressing *Snap25-GCaMP6s* with a subset marked by *tdTomato*; scale bar, 100 μm. Right: Heatmap overlay showing ΔF/F_0_ per cell following vehicle (saline), serotonin (10 μM), and KCl (50mM) stimulus. **(E)** Fraction of *tdTomato*^+^ and *tdTomato*^−^ neurons responding to each stimulus.

**Fig. 3.**
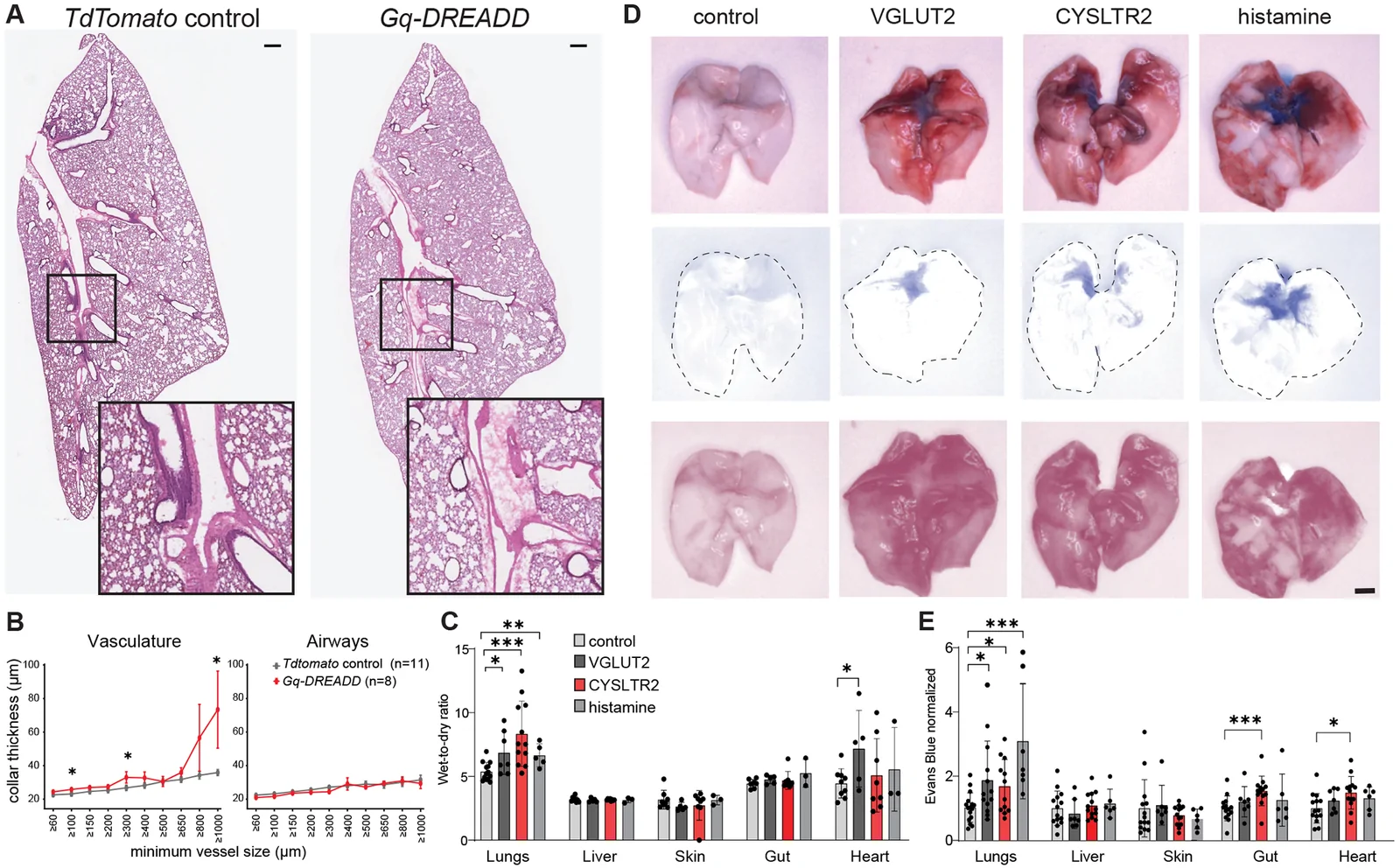
CYSLTR2 neurons induce vascular leak. **(A)** Hematoxylin and eosin (H&E)–stained lungs from *Cysltr2-ires-Cre* mice injected in the vagal ganglia with *AAV-Flex-tdTomato* (control) or *AAV-hSyn-DIO-hM3D(Gq)-mCherry* (Gq-DREADD). Bottom: higher magnification of major pulmonary vessels showing perivascular cuffing. Scale bar, 1 mm. **(B)** Collar thickness (μm) surrounding pulmonary blood vessels (left) or airways (right), binned by minimum vessel diameter, in *tdTomato* control (gray) and *Gq-DREADD* (red) mice. n = 8-11 mice per group; two-tailed unpaired Welch’s t test per bin. **(C)** Wet-to-dry ratios of lung, liver, skin, gut, and heart after optogenetic stimulation (50Hz, 5ms pulses, 10s on-20s off for 10 minutes) in *Cre*-negative *loxP-ChR2* controls (light gray), *Vglut2-Cre;loxP-ChR2* (dark gray), and *Cysltr2-Cre;loxP-ChR2* (red) mice, and in mice given endotracheal histamine (0.15 mg, medium gray). n = 3–14 mice per group; two-tailed unpaired t test. **(D)** Representative whole lung lobes (top) with Evans blue (middle) and red (bottom) channels separated by color deconvolution (see supplementary materials). Scale bar, 2 mm. **(E)** Evans blue absorbance normalized to the mean absorbance of control lobes; groups and colors and stim conditions as in (C). n = 7–14 mice per group; two-tailed unpaired t test. Asterisks as in Fig. 1D; data are means ± SEM.

**Fig. 4.**
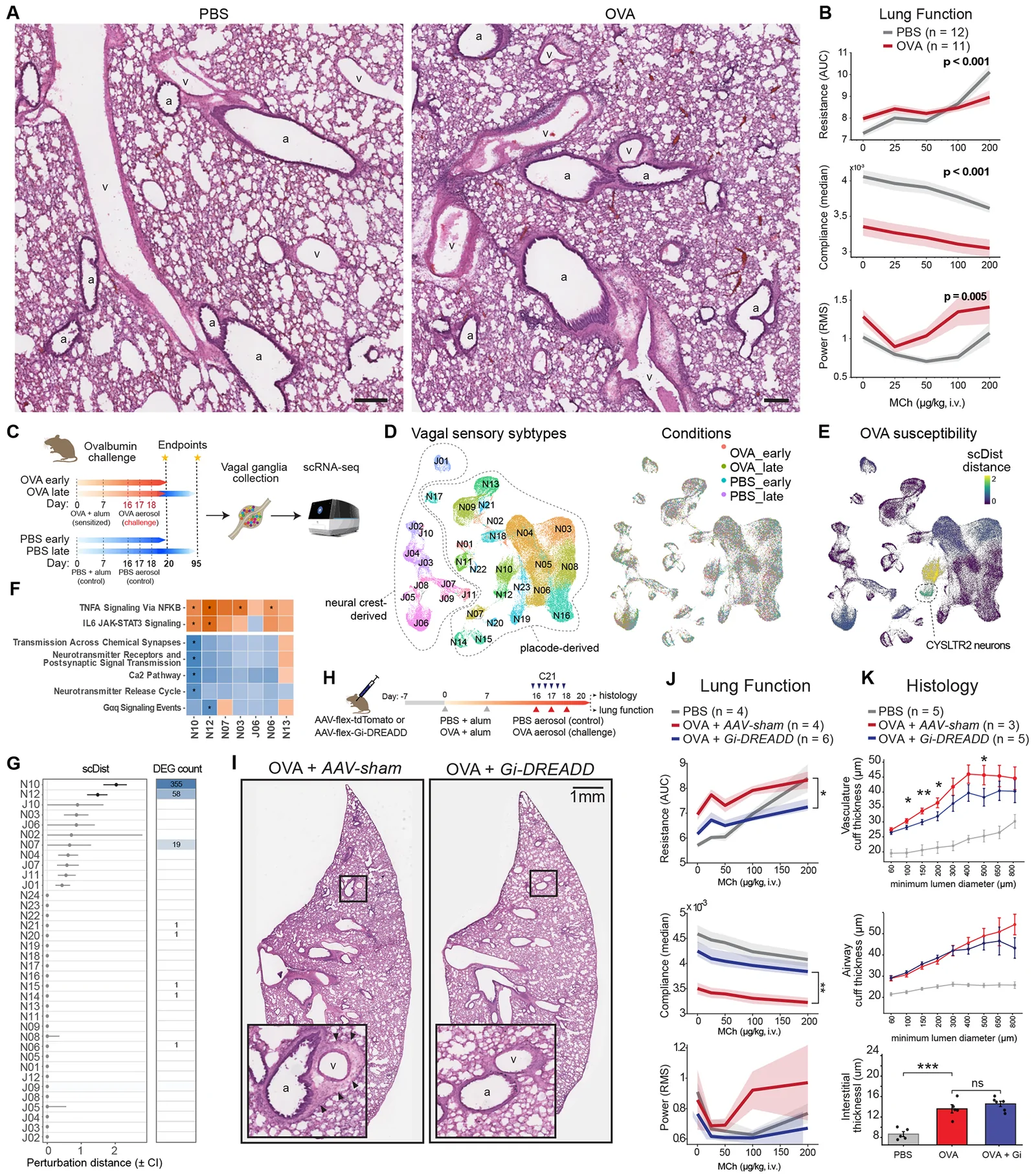
CYSLTR2 neurons respond to airway allergens and mediate allergen-evoked vascular leak in the lungs. **(A)** H&E-stained lung sections from PBS- and ovalbumin (OVA)–treated mice showing perivascular cuffing; a, airway; v, vessel. Scale bar, 250 μm. **(B)** Resistance (AUC), compliance (median), and wave power during MCh challenge (i.v.; 25, 50, 100, 200 μg/kg) in PBS-treated (gray, n = 12) and OVA-treated (red, n = 11) mice. P-values reflect omnibus model as in Fig. 1E. **(C)** Schematic of single-cell transcriptomic analysis, showing collection endpoints. **(D)** Integrated UMAP showing vagal sensory neuron subtypes, pooled across all PBS- and OVA-treated mice at early and late endpoints (20 mice per condition; 129,810 cells total). Left: jugular (J1–J10) and nodose/petrosal (N1–N23) clusters are annotated. Right: same UMAP pseudocolored by condition. **(E)** Same UMAP pseudocolored by scDist perturbation distance. **(F)** Selected enriched gene sets of OVA-altered genes in clusters N10, N12, N03, J06, N06, and N13, calculated with fgsea; colored by normalized enrichment score, asterisks indicate significant enrichment (q<0.05). **(G)** scDist perturbation distance (±95% CI) and differentially expressed gene (DEG) count per nodose/jugular population. **(H)** Schematic of timeline following intranodose injection of *AAV-Flex-tdTomato* or *AAV-hSyn-DIO-hM3D(Gi)-mCherry*, with the PBS or OVA sensitization-and-challenge. Blue arrows indicate C21 dosing (i.p., 3 mg/kg, twice daily), red arrows indicate OVA aerosol during challenge. **(I)** H&E-stained lung sections from OVA-treated *Cysltr2-ires-Cre* mice injected in the nodose ganglia with *AAV-Flex-tdTomato* (OVA + *AAV-sham*) or *AAV-hSyn-DIO-hM3D(Gi)-mCherry* (OVA + *Gi-DREADD*), with inset showing higher magnification of major pulmonary vessels (v) and airways (a); black arrows indicate cuffing. Scale bar, 1 mm. **(J)** Resistance (AUC), compliance (median), and wave power during MCh challenge (doses as in (B)) in mice treated with PBS + *AAV-Flex-tdTomato* (gray, n = 4), OVA + *AAV-Flex-tdTomato* (red, n = 4), and OVA + *AAV-hSyn-DIO-hM3D(Gi)-mCherry* (blue, n = 6). P-value reflects omnibus model, as in Fig. 1E; only the p-value for OVA + AAV-sham versus OVA + AAV-Gi-DREADD is shown. For PBS versus OVA, PBS versus OVA + Gi, and OVA versus OVA + Gi, respectively: resistance, P < 0.001, P < 0.001, P = 0.019; compliance, P < 0.001, P = 0.009, P = 0.002; wave power, P = 0.314, P = 0.439, P = 0.070. **(K)** Different histological metrics were quantified in mice treated with PBS (gray, n = 5), OVA (red, n = 3 cuff, n = 5 interstitial), and OVA + Gi (blue, n = 5 cuff, n = 6 interstitial). Top: perivascular cuff thickness, binned by minimum vessel diameter. Middle: peribronchial cuff thickness, binned by minimum airway diameter. Bottom: interstitial thickness. Welch’s unpaired t test; asterisks denote OVA versus OVA + Gi; PBS differed from both OVA and OVA + Gi at P < 0.0001 in all bins. Asterisks as in Fig. 1D; data are means ± SEM.

**Fig. 5.**
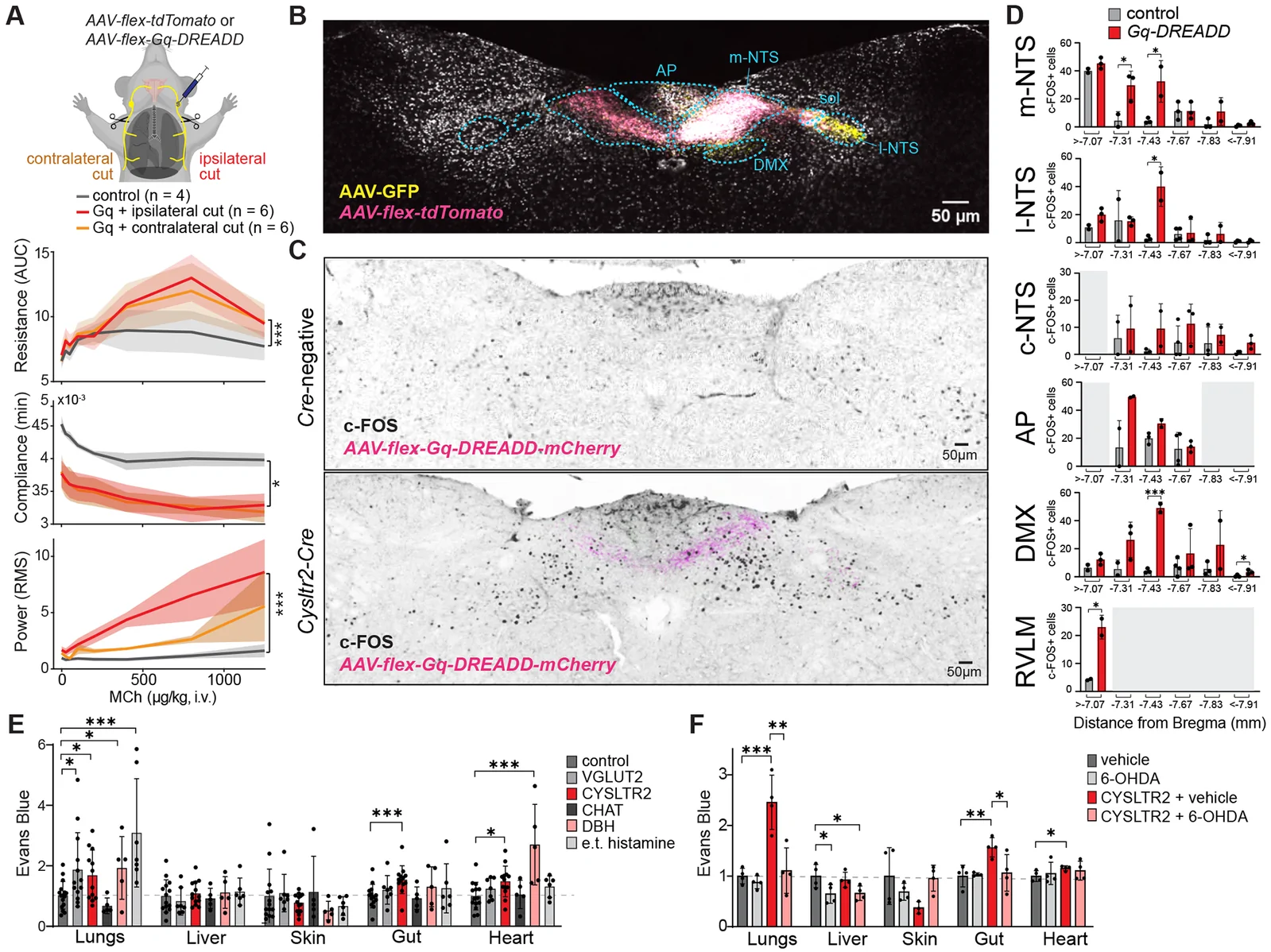
Pulmonary vascular leak is mediated by a central vagal-sympathetic reflex circuit. **(A)** Top: Schematic of unilateral CYSLTR2 neuron activation combined with ipsilateral or contralateral vagotomy. Bottom: resistance (AUC), compliance (median), and wave power (AUC RM during MCh challenge (i.v.; 25, 50, 100, 200, 400, 800, 1250 μg/kg) in *Cysltr2-ires-Cre* mice injected unilaterally with *AAV-Flex-tdTomato* (gray, n = 4), unilaterally with *AAV-hSyn-DIO-hM3D(Gq)-mCherry* with ipsilateral vagotomy (red, n = 6), or unilaterally with *AAV-hSyn-DIO-hM3D(Gq)-mCherry* with contralateral vagotomy (orange, n = 6). P-values reflect omnibus model, as in Fig. 1E. For control versus Gq-ipsilateral, control versus Gq-contralateral, and Gq-ipsilateral versus Gq-contralateral, respectively: resistance, P = 0.002, P = 0.199, P = 0.948; compliance, P = 0.044, P = 0.076, P = 0.907; wave power, P < 0.001, P = 0.003, P = 0.336. Asterisks denote control versus Gq-ipsilateral. **(B)** A brainstem section from a *Cysltr2-ires-Cre* mouse unilaterally injected with *AAV-Flex-tdTomato* and *pENN.AAV.CB7.CI.eGFP.WPRE.rBG*. Scale bars, 50 μm; bregma −7.43 mm. **(C)** c-Fos (black) and mCherry (magenta) immunostaining in *Cre*-negative and *Cysltr2*-ires-*Cre* mice injected with *AAV-hSyn-DIO-hM3D(Gq)-mCherry*, 90 minutes after i.p. C21. Scale bar, 50 μm. **(D)** c-Fos^+^ neuron counts in m-NTS, l-NTS, c-NTS, AP, DMV, and RVLM, binned by anatomical level (distance from bregma), n = 2–4 mice; two-tailed unpaired t test. **(E)** Vascular leak was quantified by Evans blue absorbance after optogenetic stimulation (5ms pulses, 10s on/20s off for 10 minutes) of *Cre*-negative controls (gray; 50Hz), *Vglut2-Cre* (light gray; 50Hz), *Cysltr2-Cre* (red; 50Hz), *Chat-Cre* (dark gray; 10Hz), or *Dbh-Cre* (pink; 10Hz) neurons. Values for each organ were normalized to the mean absorbance of controls. Endotracheal histamine (0.15 mg, grey) was provided as a positive control. n = 7–14 mice per group; two-tailed unpaired t test. **(F)** Evans blue absorbance normalized to the mean of vehicle control lobes in vehicle-treated (gray) and 6-OHDA–treated (light gray) mice, and in *Cysltr2-Cre;loxP-ChR2* mice given vehicle (red) or 6-OHDA (pink). All mice were optogenetically stimulated using same parameters as in (E). n = 4 mice per group; two-tailed unpaired t test. Asterisks as in Fig. 1D; data are means ± SEM. AP, area postrema; DMV, dorsal motor nucleus of the vagus; NTS, nucleus of the solitary tract (m-, medial; l-, lateral; c-, commissural); RVLM, rostral ventrolateral medulla.
