# A vagal-to-sympathetic reflex controls lung vascular leak

**DOI:** 10.64898/2026.10.22.761052

**Published:** 2026-11-03

**Authors:** Emily K. Robinson, Emma M. Ibanez, Blanca Santibanez Ocampo, Juan Li, Nupur Ballal, Joelle M. Miller, Kaustubh Shivdikar, Benjamin D. Umans, Sara L. Prescott

## Abstract

Dynamic regulation of the vascular barrier is required for innate immunity, yet excessive leak endangers vulnerable organs like the lungs, where edema stiffens tissue and threatens breathing. Using a chemogenetic screen, we discover a vagal-to-sympathetic reflex that controls pulmonary vascular leak. The reflex originates in a previously-uncharacterized population of polymodal, alveolus-innervating vagal sensory neurons marked by *Cysltr2* . Stimulating CYSLTR2 neurons increases lung fluid within minutes, whereas sustained activation elicits perivascular edema and respiratory dysfunction. Silencing them during allergen challenge reduces leak and improves lung mechanics. Rather than acting locally, CYSLTR2 neurons relay centrally to sympathetic efferents to broadcast effects throughout lung vasculature. This pathway thus forms an axis of organ defense that can go awry in disease, suggesting new ways to control lung fluid balance.

