# Supplementary material for: A vagal-to-sympathetic reflex controls lung vascular leak
Source: bioRxiv. 2026 Oct 27:2026.10.22.761052. Preprint. [Version 1] doi: 10.64898/2026.10.22.761052 (PMC13733677; doi:10.64898/2026.10.22.761052)
Supplement: Supplementary file 1 [file NIHPP2026.10.22.761052v1-supplement-4.pdf]

bioRxiv preprint doi: https://doi.org/10.64898/2026.10.22.761052; this version posted October 27, 2026. The copyright holder for this preprint (which was not certified by peer review) is the author/funder, who has granted bioRxiv a license to display the preprint in perpetuity. It is made available under a CC-BY 4.0 International license.

List of Supplementary Materials:

Materials and methods

Table S1

Table S2

Table S3

Fig S1

Fig S2

Fig S3

Fig S3

Fig S4

Fig S5

Fig S6

Fig S7

Movie 1

Movie 2

Link to Github page: https://github.mit.edu/Prescott-Lab/bumans_OVA_manuscript

bioRxiv preprint doi: https://doi.org/10.64898/2026.10.22.761052; this version posted October 27, 2026. The copyright holder for this preprint (which was not certified by peer review) is the author/funder, who has granted bioRxiv a license to display the preprint in perpetuity. It is made available under a CC-BY 4.0 International license.

Supplementary Figure Legends

Fig. S1. Genetic access to lung-innervating vagal sensory populations, and validation of airway mechanics across age and sex.

(A) UMAP showing predicted lung afferents based at least one of three published datasets[3,13,16]. **(B)** UMAPs showing marker gene expression across vagal sensory neuron subsets. **(C)** Top: larynx whole-mount preparations comparing *Calb1-ires-Cre* and *Kcng4-Cre* tracing. Bottom: higher magnification of laryngeal terminals. **(D)** Resistance (AUC), compliance (median), and wave power during MCh challenge (i.v.; 25, 50, 100, 200 μg/kg). Left: by age, 8 to 9 weeks (yellow, n = 25), 10 to 11 weeks (orange, n = 9), and ≥12 weeks (red, n = 10). Right: by sex, male (green, n = 5) and female (blue, n = 5). Omnibus model with dose as a categorical factor, group × dose interaction reported. **(E)** Change in respiratory rate and tidal volume during vagal optogenetic stimulation in freely breathing mice expressing *ChR2* from the indicated *Cre* drivers. n = 3–4 mice per line; two-tailed unpaired t test; $^{*}P < 0.05$, $^{**}P < 0.01$, $^{***}P < 0.001$, $^{****}P < 0.0001$. Data are means ± SEM.

bioRxiv preprint doi: https://doi.org/10.64898/2026.10.22.761052; this version posted October 27, 2026. The copyright holder for this preprint (which was not certified by peer review) is the author/funder, who has granted bioRxiv a license to display the preprint in perpetuity. It is made available under a CC-BY 4.0 International license.

Fig. S2. Cysltr2$^+$ vagal neuron projection sites outside the lungs.

(A) Confocal images of *Cysltr2*$^+$ vagal fibers in the indicated organs (300-μm cryosections, anti-tdTomato immunostaining) from *Cysltr2-ires-Cre* mice injected in the nodose ganglia with *AAV-flex-tdTomato*. Scale bars are indicated for each image. **(B)** *Cysltr2*+ fibers (black) innervate neuroepithelial bodies (green) marked by blue arrows. Scale bars, 10 μm. **(C)** Representative image showing FISH labeling on a 14 μm section of nodose for *ChR2* (green) and *Cysltr2* (magenta) probes manufactured by Molecular Instruments. Scale bar, 100 μm. **(D)** Venn diagram shows the number of cells expressing either probe alone or both probes, quantifying the overlap in expression at the single-cell level. **(E)** Heatmap of $\Delta F/F_0$ over time for tdTomato$^+$ (22 out of 22 cells) and tdTomato- neurons (11 out of 39) during sequential perfusion of LTC_4 and LTD_4 cysteinyl leukotriene cocktail, capsaicin, and KCl and ranked by capsaicin response.

Fig. S3. *Cysltr2*$^+$ vagal neuron activation selectively affects pulmonary vascular physiology.

(A) Nuclear staining, protein staining, septal thickness, alveolar size, cell density, goblet cell hyperplasia, and immune cell infiltration in *Cysltr2-ires-Cre* mice injected in the NJP ganglia with *AAV-Flex-tdTomato* (control) or *AAV-hSyn-DIO-hM3D(Gq)-mCherry* (Cysltr2-Gq). n = 3–8 mice per group; Welch's two-tailed unpaired t test. **(B)** Representative tracheal pressure (left) and heart rate EMG (right) traces during optogenetic stimulation (yellow overlay) in *Cre*-negative, *Vglut2-Cre;loxP-ChR2*, and *Cysltr2-Cre;loxP-ChR2* mice. **(C)** Respiratory rate, tidal volume, resistance, compliance, and heart rate for the indicated *Cre* lines. n = 3–4 mice per line; two-tailed unpaired t test; asterisks as in Fig. S1E. Data are means ± SEM.

Fig. S4. Extended single-cell transcriptomic analysis of vagal sensory neurons during allergic inflammation.

bioRxiv preprint doi: https://doi.org/10.64898/2026.10.22.761052; this version posted October 27, 2026. The copyright holder for this preprint (which was not certified by peer review) is the author/funder, who has granted bioRxiv a license to display the preprint in perpetuity. It is made available under a CC-BY 4.0 International license.

(A) UMAP showing cell type identity for all cells collected for scRNA-seq. **(B)** Cluster correspondence matrix comparing de novo clusters (y axis) with Prescott et al. clusters (x axis); black indicates transcriptionally matched pairs. Each de novo subtype mapped to a single published cluster. **(C)** Cell counts per de novo cluster, split by condition (PBS early, OVA early, PBS late, OVA late) and total, with the percentage of each group's total indicated. **(D)** DEG counts per cluster at day 20 and day 95. **(E)** Resistance (AUC), compliance (median), and wave power during MCh challenge (i.v.; 25, 50, 100, 200, 400 µg/kg) in PBS chase (green, n = 3) and OVA chase (blue, n = 3) mice. P-values calculated with omnibus model as in Fig. S1D. **(F)** UMAPs showing expression of the indicated genes. **(G)** Dot plot showing the percentage of cells expressing each gene and mean expression level across vagal sensory subtypes. **(H)** Proportion of tdTomato^{+} neurons responding to Yoda1 and, among those, the proportion responding to bradykinin or capsaicin; n and percentages are shown on each chart. **(I)** Heat map of $\Delta F/F_0$ over time for tdTomato^{+} neurons during sequential perfusion of Yoda1, bradykinin, capsaicin, and KCl. Data are means ± SEM.

Fig. S5. Silencing *Cysltr2*$^{+}$ neurons rescues lung function and structure during severe allergic challenge.

(A) OVA-specific and total IgE (µg/mL) in serum collected at day 20 from saline controls (gray), OVA-treated *Cysltr2-ires-Cre* mice injected in the nodose with *AAV-Flex-tdTomato* (pink), and OVA-treated *Cysltr2-ires-Cre* mice injected with *AAV-hSyn-DIO-hM3D(Gi)-mCherry* (blue). The same cohorts and colors apply to (B) to (F). n = 6-12 mice per group; Welch's unpaired t test. **(B)** UEA-1^{+} area normalized to bronchial area in lung cryosections collected at days 18, 20, and 27 (4 to 6 lobes per mouse; n = 3–5 mice per group); two-tailed unpaired t test. **(C)** Total nuclei counts (nuclei per mm^2) for the same cohorts at days 18, 20, and 27. n = 3-6; Welch's unpaired t test. (**D**) Pressure-volume loops for each cohort. **(E)** Extended vascular and airway cuff thickness at days 18, 20, and 27. n = 3-6; Welch's unpaired t test. **(F)** (Top) Septal/Interstitial

bioRxiv preprint doi: https://doi.org/10.64898/2026.10.22.761052; this version posted October 27, 2026. The copyright holder for this preprint (which was not certified by peer review) is the author/funder, who has granted bioRxiv a license to display the preprint in perpetuity. It is made available under a CC-BY 4.0 International license.

thickness from H&E sections at days 18, 20, and 27. (Bottom) Alveolar size measured using mean linear intercept (μm) from H&E sections at days 18, 20, and 27; lobes were inflation-normalized as described in the supplementary materials. n = 3-6; Welch's unpaired t test. Asterisks as in Fig. S1E; data are means ± SEM.

Fig. S6. Characterization of neural pathways regulating pulmonary vascular fluid flux. **(A)** Resistance (AUC), compliance (median), and wave power during MCh challenge (i.v.; 25, 50, 100, 200, 400, 800, 1250 μg/kg) in *AAV-Flex-tdTomato*-injected controls (gray, n = 4) and *Cysltr2-ires-Cre* mice with unilateral *AAV-hSyn-DIO-hM3D(Gq)-mCherry* injection (red, n = 3). Omnibus model as in Fig. S1D; data are means ± SEM. **(B)** GFP and RFP immunostaining in *Cysltr2-ires-Cre* mice unilaterally injected with *pENN.AAV.CB7.CI.eGFP.WPRE.rBG* and *AAV-Flex-tdTomato*. **(C)** c-FOS immunostaining in serial brainstem sections 90 minutes after C21 in *Cre*-negative controls (left) and *Cysltr2-ires-Cre* mice (right). c-FOS$^+$ cells were enriched in the m-NTS from bregma −7.31 to −7.43 mm. Outlines delineate NTS subregions at the indicated bregma levels. Scale bars, 50 μm. **(D)** Top: TH (yellow) and CHAT (magenta) immunostaining in a whole-mount lung slice (500 μm); nuclei, DAPI (blue). Blood vessels indicated on image. Scale bar, 500 μm.

Fig. S7. Proposed circuit by which CYSLTR2$^+$ vagal sensory neurons drive sympathetically mediated pulmonary vascular leak.

Table S1. Primary and Secondary Antibodies for Immunostaining.

A = Alexa Fluor dye conjugate.

bioRxiv preprint doi: https://doi.org/10.64898/2026.10.22.761052; this version posted October 27, 2026. The copyright holder for this preprint (which was not certified by peer review) is the author/funder, who has granted bioRxiv a license to display the preprint in perpetuity. It is made available under a CC-BY 4.0 International license.

**Antibody Target**	**Host Species**	**Dilution**	**Catalog No.**	**Supplier**	**Application**
Primary Antibodies					
RFP	Chicken	1:1000	600-901-379	Rockland	Lung tracing; Brain
CD31	Goat	1:200	AF3628	R&D Systems	Lung tracing
GFP	Rabbit	1:1000	RPCA-GFP	EnCor Biotech	Brain and Ganglia sections
mCherry	Chicken	1:1000	CPCA-mCherry	EnCor Biotech	Brain and Ganglia sections
UEA-1 Conjugated FITC (488)	N/A (lectin)	1:1000	FL-1061	Vector Laboratories	Lung Epithelial histology
CD45	mouse	1:500	157602	Biolegend	Lung histology
c-Fos	Rat	1:1000	226017	SYSY	Brain, SCG, stellate sections

bioRxiv preprint doi: https://doi.org/10.64898/2026.10.22.761052; this version posted October 27, 2026. The copyright holder for this preprint (which was not certified by peer review) is the author/funder, who has granted bioRxiv a license to display the preprint in perpetuity. It is made available under a CC-BY 4.0 International license.

Secondary Antibodies					
Anti-Goat (A647)	Donkey	1:500	705-605-003	Jackson ImmunoResearch	Lung tracing
Anti-Chicken (A555)	Donkey	1:300	703-565-155	Jackson ImmunoResearch	Lung tracing; ganglia; Brain sections
Anti-Rabbit (A488)	Donkey	1:300	711-545-152	Jackson ImmunoResearch	Lung tracing; ganglia; Brain sections
Anti-Rat (A647)	Donkey	1:300	712-605-150	Jackson ImmunoResearch	Brain, SCG, stellate sections
Anti-Mouse (A647)	Donkey	1:300	715-607-003	Jackson ImmunoResearch	Lung histology

Table S2: Color Deconvolution Settings

**Color 1 (Evans Blue)**	**Color 2 (red-blood)**	**Color 3 (green)**

bioRxiv preprint doi: https://doi.org/10.64898/2026.10.22.761052; this version posted October 27, 2026. The copyright holder for this preprint (which was not certified by peer review) is the author/funder, who has granted bioRxiv a license to display the preprint in perpetuity. It is made available under a CC-BY 4.0 International license.

0.69890785	0.37861538	0.41732463
0.64401823	0.7191889	0.7968193
0.31107616	0.58259565	0.43694293

Table S3: Combined GSEA across all vagal subtypes. Significant gene set enrichment analysis (GSEA) results for N10 and N12, analyzed across all vagal subtypes.

Movie S1. Three-dimensional reconstruction of CYSLTR2+ vagal sensory neuron projections in the pulmonary alveolar parenchyma. Three-dimensional rendering of virally labeled CYSLTR2+ vagal sensory neuron fibers (white) extending around a major conducting airway labeled with smooth muscle actin (green) and projecting into the alveolar parenchyma. CD31+ blood capillaries are labeled in purple, and nuclei are labeled with DAPI (blue).

Movie S2. High magnification three-dimensional reconstruction of CYSLTR2+ vagal sensory neuron projections in the alveolar parenchyma. Three-dimensional rendering of virally labeled CYSLTR2+ vagal sensory neuron fibers (white) innervating the alveolar parenchyma, with CD31+ blood capillaries labeled in purple and nuclei labeled with DAPI (blue). The imaged region corresponds to that shown in Fig. 2B.

Material & Methods

Mice

All animal husbandry and procedures were performed in compliance with institutional animal care and use committee guidelines. Age-matched 8-11 week-old male littermate mice were used for all experiments unless otherwise noted. The following mouse transgenic lines were used: *Gabra1-ires-Cre* (gift from the Liberles lab, Harvard Medical School); *Npy1r-Cre* (Jackson

bioRxiv preprint doi: https://doi.org/10.64898/2026.10.22.761052; this version posted October 27, 2026. The copyright holder for this preprint (which was not certified by peer review) is the author/funder, who has granted bioRxiv a license to display the preprint in perpetuity. It is made available under a CC-BY 4.0 International license.

Laboratory, 030544); *Kcng4-Cre* (Jackson Laboratory, 029414); *Vglut2-ires-Cre* (Jackson Laboratory, 029414); *Calb1-ires2-Cre* (Jackson Laboratory, 028532)*; Cysltr2-ires-Cre* (gift from the Isaac Chiu, Harvard Medical School); *Piezo2-eGFP-ires-Cre* (Jackson Laboratory, 027719), and *Vip-Cre* (Jackson Laboratory, 010908). Reporter/effector lines include *Ai14* (*loxP-tdTomato*; Jackson Laboratory, 007908), *Ai32* (*loxP-ChR2*; 012569), and *loxP-DTR* (Jackson Laboratory, 007900). Calcium imaging was performed on neurons from *Snap25-2A-GCaMP6s* mice (B6.Cg-Snap25tm3.1Hze/J; Jackson Laboratory, 025111), which express the genetically encoded calcium indicator GCaMP6s pan-neuronally from the endogenous *Snap25* locus, crossed to *Cysltr2-ires-Cre* and a *Cre*-dependent *Rosa26-loxP-tdTomato* reporter so that *Cysltr2*-lineage neurons could be identified by red fluorescence within the same imaging field. Wildtype controls were C57BL/6J (Jackson Laboratory, 000664).

Ovalbumin (OVA) challenge

Male mice aged 8-11 weeks were sensitized by intraperitoneal (i.p.) injection of ovalbumin (OVA; 50μg/mouse; Sigma-Aldrich, A5503) with aluminum hydroxide adjuvant (1.6mg/mouse; Alhydrogel 2%, InvivoGen) on days 0 and 7. On days 16–18, mice were exposed to aerosolized 1% OVA (2 mL total volume) in 1 L/min oxygen flow for 30 minutes during the light cycle. Control animals received aluminum hydroxide adjuvant (1.6mg/mouse; Alhydrogel 2%, InvivoGen, i.p.) and saline on days 0 and 7, then exposed to aerosolized saline with 1 L/min oxygen flow for 30 minutes on days 16–18. Animals were anesthetized for lung function testing or euthanized for tissue harvest at day 20 unless otherwise specified.

Methacholine challenge and lung function testing

Mice were anesthetized with tribromoethanol (375 mg/kg). Upon reaching a surgical plane of anesthesia, animals were tracheostomized, and mechanically ventilated (SAR-1000, CWE) at

bioRxiv preprint doi: https://doi.org/10.64898/2026.10.22.761052; this version posted October 27, 2026. The copyright holder for this preprint (which was not certified by peer review) is the author/funder, who has granted bioRxiv a license to display the preprint in perpetuity. It is made available under a CC-BY 4.0 International license.

150 breaths/min with a tidal volume of 7.5 mL/kg. Each ventilated breath was programmed with 120ms for inspiration, 80ms breath-hold maneuver, and 200ms for the expiration. The diaphragm was transected to eliminate spontaneous breathing. Positive end-expiratory pressure was set to 20mmH_2O and 100% oxygen was supplemented during ventilation. Tracheal pressure was measured by connecting a tracheal cannula port to a differential pressure transducer. Airflow was measured using a pneumotachograph (Harvard Apparatus, 73-0981) connected to a differential pressure transducer (Harvard Apparatus, 73-0064). Data from both transducers was amplified (BIOPAC, DA100C) and recorded continuously at 2 kHz using AcqKnowledge Software (BIOPAC).

For methacholine bronchoprovocation assays, mice were given methacholine every two minutes after a 1 minute baseline at a rate of 0.2mL/min (Doses: 25, 50, 100, 200, 400, 800, 1250 mg/kg; USP) via retro-orbital injection.

Airway resistance and compliance measurements

Respiratory mechanics were computed breath by breath with custom MATLAB code (MathWorks) using the constant-flow, end-inspiratory-occlusion formulation established for mechanically ventilated subjects[76]. Briefly, breath boundaries were defined from the volume signal. Airflow was smoothed with a third-order Savitzky-Golay filter, corrected for slow baseline drift by subtraction of a running median, and integrated to yield volume. Inspiratory and expiratory volume extrema marked the ends of inspiration and expiration, and the mode of the kernel density estimate of peak prominences across the recording defined the average tidal volume used to set detection thresholds. Three pressure landmarks were then identified per breath. Peak inspiratory pressure (PIP) was taken as the maximum tracheal pressure between successive end-inspiratory volume peaks, refined to the local maximum within a 20-sample window. The end-inspiratory plateau pressure (Pplat) and the end-expiratory pressure (PEEP)

bioRxiv preprint doi: https://doi.org/10.64898/2026.10.22.761052; this version posted October 27, 2026. The copyright holder for this preprint (which was not certified by peer review) is the author/funder, who has granted bioRxiv a license to display the preprint in perpetuity. It is made available under a CC-BY 4.0 International license.

were taken from the first and second inflections of the second derivative of the filtered pressure trace following PIP. Breaths whose PIP was an outlier relative to a 20-breath moving mean, and breaths whose PIP-to-plateau interval exceeded twice the median interval, were excluded.

Airway resistance (Raw) for each breath was calculated as the pressure drop across the resistive component divided by the inspiratory flow,

$$R_{aw} = \frac{P_{PIP} - P_{plat}}{\dot{V}} - R_{tubing}$$

where $\dot{V}$ is the constant inspiratory flow in mL/min. The resistance of the cannula and ventilator tubing was measured in the absence of an animal (0.0101 mmH_2O per mL/min) and subtracted from every resistance value. Quasi-static compliance was calculated as the delivered tidal volume divided by the elastic pressure drop,

$$C = \frac{\dot{V}\, t_{insp}}{P_{plat} - P_{PEEP}}$$

where t_{insp} is the fixed inspiratory flow time (taken as the interval from end-expiration to PIP), so that the product $\dot{V} t_{insp}$ is the volume delivered during inspiratory flow. Resistance is reported in mmH_2O/(mL/min) and compliance in mL/mmH_2O. For each methacholine dose, the 60-s window beginning at the time of delivery was analyzed, and the area under the resistance curve and the median compliance within that window are reported.

To quantify breath-to-breath instability of airway resistance, we performed a sliding-window spectral analysis of the detrended breath-by-breath resistance signal. A robust locally weighted trend (rlowess, span 5 breaths) was subtracted from this series to yield resistance residuals, isolating breath-to-breath fluctuations from the slow underlying trend. The residual series was then transformed into the time–frequency domain by a short-time Fourier transform

bioRxiv preprint doi: https://doi.org/10.64898/2026.10.22.761052; this version posted October 27, 2026. The copyright holder for this preprint (which was not certified by peer review) is the author/funder, who has granted bioRxiv a license to display the preprint in perpetuity. It is made available under a CC-BY 4.0 International license.

(spectrogram; 30-breath Hamming window, 29-breath overlap, evaluated at the breath-series sampling rate), and the power spectrum was taken as the squared magnitude of the transform. At each time bin, a spectral root-mean-square (RMS) amplitude was calculated as the square root of the mean spectral power across all frequency bins, giving a breath-indexed trace of the RMS amplitude of the resistance residuals. For each methacholine dose, a 60-s analysis window beginning at that dose's marker time was defined, and "RMS (AUC)" was computed as the trapezoidal integral of the spectral RMS trace over this window (time in seconds), yielding one cumulative RMS value per dose per animal that was used as the summary response in subsequent dose–response analyses.

Dose-response curves were compared between groups with a linear mixed-effects model with mouse as a random intercept, fit by maximum likelihood. An omnibus *P* value was obtained by likelihood-ratio comparison of Response ~ Group × Dose + (1|Mouse) against Response ~ Dose + (1|Mouse), with dose treated as a categorical factor. This test detects a difference in overall level, in curve shape, or both. Curves show group mean ± SEM (n mice per group given in each figure).

Tissue collection and immunofluorescence

Mice were euthanized with pentobarbital and then cardiac perfused with ice-cold 4% paraformaldehyde (PFA) in saline. Lungs were inflated with 1mL of cold 4% PFA before dissection through the trachea at a rate of 1mL/min. Tissues were post-fixed overnight in 4% PFA at 4°C. Brains and lungs were cryoprotected in 30% sucrose, incubated in O.C.T. for 1 hour before freezing and sectioning.

For lung tracing experiments, thick (300-µm) cryosections were stained with primary and secondary antibodies as listed in Table S1 for 16-36 hours at 4°C. Samples were cleared using EasyIndex (LifeCanvas Technologies) and imaged on a Leica Stellaris confocal microscope.

bioRxiv preprint doi: https://doi.org/10.64898/2026.10.22.761052; this version posted October 27, 2026. The copyright holder for this preprint (which was not certified by peer review) is the author/funder, who has granted bioRxiv a license to display the preprint in perpetuity. It is made available under a CC-BY 4.0 International license.

Brain sections (40μm) were stained with primary and secondary antibodies as listed in Table S1.

Ganglia were processed using the EZ Clear protocol[77] and stained with primary antibodies as listed in Table S1.

Serum IgE quantification by ELISA

Total and OVA-specific IgE were measured in serum collected at day 20 of the challenge protocol. Mice were anesthetized with avertin (tribromoethanol; 375 mg/kg i.p.), the descending aorta was transected, and approximately 1 mL of blood was collected into serum separator tubes. Samples were held on ice until collection was complete for all animals in the cohort, then allowed to clot for 45 minutes before being centrifuged at 2,000 × g for 15 min at 4 °C. Serum was transferred to clean tubes and stored at −80 °C until assay, with no more than one freeze–thaw cycle.

Total IgE was measured with Mouse Total IgE ELISA Kit (RayBio Tech, ELM-IgE-1) and OVA-specific IgE with LEGEND MAX™ Mouse OVA Specific IgE ELISA Kit (Biolegend, 439807) following the manufacturer's instructions. Samples were assayed in duplicate at [1:100] (total IgE) and [1:50] (OVA-specific IgE); dilutions chosen so that readings fell within the linear portion of the standard curve. Standards were run in duplicate across 7 serial dilutions on every plate. Absorbance was read at 450 nm on a ThermoFisher Varioskan Flash plate reader, and concentrations were interpolated from a four-parameter logistic fit. The limit of detection was 20.7 pg/mL (for Anti-OVA IgE ELISA) and 0.14ng/mL(for Total IgE ELISA); samples reading below it were assigned the limit of detection. Where samples were distributed across more than one plate, a pooled reference serum was included on every plate and used for normalization.

Nodose ganglion injections and vagotomy

bioRxiv preprint doi: https://doi.org/10.64898/2026.10.22.761052; this version posted October 27, 2026. The copyright holder for this preprint (which was not certified by peer review) is the author/funder, who has granted bioRxiv a license to display the preprint in perpetuity. It is made available under a CC-BY 4.0 International license.

Animals were anesthetized using isoflurane (1-3%, delivered in oxygen) and placed in a supine position. Vagal ganglion injections were performed as previously described[3,5]. AAVs were mixed with Fast Green dye (10:1 ratio, to 0.1% final dye concentration) and injected into vagal ganglia in a volume of 140nl, using a Nanoject II injector (Drummond), and success determined visually by Fast Green Dye filling of the ganglia. Tracing experiments used a 1:1 titer ratio of *pAAV-Flex-tdTomato*(Addgene 28306-PHP.S, v247992, 1.9 x 10^13 gc/mL) and *pENN.AAV.CB7.CI.eGFP.WPRE.rBG* (Addgene 105542-AAV9, v36906, 2.3x10^13 gc/mL). Chemogenetic experiments used *AAV-hSyn-DIO-hM3D(Gq)-mCherry* (Addgene 44361-AAV9, v227973, 1.5x10^13 gc/mL) or *AAV-hSyn-DIO-hM3D(Gi)-mCherry* (Addgene 44362-AAV9, v241463, 1.9x10^13 gc/mL) viruses or *pAAV-Flex-tdTomato* virus for control experiments.

For vagotomy experiments, the vagus nerve was exposed following the same procedure as used for nodose ganglion injections. The vagal trunk was then resected from surrounding tissue, transected approximately 1mm below the SLN branchpoint and cauterized following unilateral nodose injection.

Chemogenetic vagal manipulation

Following ganglion injection of *AAV-hSyn-DIO-hM3D(Gq)-mCherry* or *AAV-hSyn-DIO-hM3D(Gi)-mCherry* viruses, mice recovered for 1 week before initiating OVA sensitization. Compound 21 (C21; 3 mg/kg) was administered via intraperitoneal injection twice daily (8-hour interval) on days 16–18, on concurrent days with the OVA aerosol challenge. Methacholine measurements were performed on day 20. Additionally, D18 (2 hours after last C21 dose) and D27 timepoints were used for lung structure analyses.

Brainstem tracing and c-Fos mapping

For brainstem tracing, male *Cysltr2-ires-Cre* mice received unilaterally injections of a viral tracing cocktail containing *pAAV-Flex-tdTomato* and *pENN.AAV.CB7.CI.eGFP.WPRE.rBG* (1:1)

bioRxiv preprint doi: https://doi.org/10.64898/2026.10.22.761052; this version posted October 27, 2026. The copyright holder for this preprint (which was not certified by peer review) is the author/funder, who has granted bioRxiv a license to display the preprint in perpetuity. It is made available under a CC-BY 4.0 International license.

into the nodose ganglion, as described above. Mice were allowed to recover for 4 weeks before pentobarbital euthanasia, followed by tissue collection and sectioning as described above.

For c-Fos mapping experiments, male *Cysltr2-ires-Cre* mice (*Cysltr2*-Gq) and C*re*-negative littermate controls received injections of *AAV-hSyn-DIO-hM3D(Gq)-mCherry* into the nodose ganglion. Mice were allowed to recover for 4 weeks before intraperitoneal administration of C21 (3 mg/kg). Ninety minutes after C21 administration, mice were euthanized with pentobarbital overdose, and tissues were collected as described above. Brainstem sections were co-stained with antibodies against c-Fos and mCherry, provided in Table S1.

Single-cell RNA sequencing

Nodose/jugular ganglia were harvested from 80 adult male C57BL/6J mice in two batches, corresponding to days 20 and 95 after initial ovalbumin exposure. In each batch, four replicate samples of ovalbumin- or PBS-treated animals, each consisting of ganglia pooled from five animals, were collected. Each pooled sample was dissociated in 1 mL of 1:1 Dulbecco's Modified Eagle's Medium/Ham's F12 media (DMEM; Thermo Fisher Scientific, 11039047) supplemented with Liberase TH (0.22 mg/mL) and DNase (100 U/mL) at 37°C for 30 min. Ganglia were triturated with a P200 tip, filtered through a 70 μm cell strainer and pelleted (300 × g, 10 min, 4°C). Cells were fixed overnight according to GEM-X Flex protocols (10x Genomics, CG000782) and stored at -80˚C. Fixed cells were processed for sequencing in a single multiplexed batch using the 10X Genomics Flex v2 platform, according to manufacturer instructions. Libraries were sequenced on an Illumina NovaSeq X platform.

Fastq files were aligned and samples were demultiplexed using cellranger software. After processing with CellBender, feature barcode matrices were preprocessed and filtered to exclude cells with fewer than 200 UMIs. Coarse clustering (resolution 0.3) was used to isolate sensory neuron populations and detect doublets using scDblFinder[78], and nodose and jugular

bioRxiv preprint doi: https://doi.org/10.64898/2026.10.22.761052; this version posted October 27, 2026. The copyright holder for this preprint (which was not certified by peer review) is the author/funder, who has granted bioRxiv a license to display the preprint in perpetuity. It is made available under a CC-BY 4.0 International license.

sensory neurons, identified by cluster-wide expression of *Phox2b* or *Prdm12*, were independently subclustered (resolutions 0.35 and 0.2, respectively) using Seurat (v5)[79] prior to further analysis. Data were compared to prior vagal single-cell datasets using Seurat's MapQuery function. Treatment-responsive cell populations were identified using scDist[80], modeling ovalbumin vs. PBS treatment status as a fixed effect and replicate sample as a random effect. Benjamini-Hochberg false discovery rate estimates were used to identify significantly altered subtypes (N10 and N12 only). Separately, we identified differentially-expressed genes in each cell cluster independently, using DeSeq2[81] to model pseudobulk expression (calculated for each sample/cluster combination) as a function of treatment condition. In both scDist and DeSeq2 analyses, we compared ovalbumin- and PBS-treated samples at the early and late time points independently to avoid potential confounding influences of age or sample collection batch. The fgsea package[82] was used to test for enrichment of gene sets obtained from MSigDB (Hallmark, GO-Biological Processes, Reactome). Gene sets with fewer than 15 or more than 500 genes expressed in a subtype were excluded, and the Wald statistic obtained from differential expression testing was used to rank genes for enrichment testing.

Optogenetics

Mice were anesthetized with urethane (2 mg/g) for spontaneous breathing experiments or tribromoethanol (375 mg/kg) for ventilated conditions. The nodose/jugular ganglion was exposed, and an optic fiber (400 μm core; Thorlabs) was positioned over the vagal trunk. Light stimulation (10–50 Hz, 5-ms pulses, 10–30 s duration) was delivered via a DPSS laser system (473 nm, 150 mW, Ultralaser). Heart rhythm was measured via EKG, which was recorded with two needle electrodes placed subcutaneously on the right forepaw and left hindpaw and amplified with a differential amplifier (A-M Systems), with a baseline needle in the right hindpaw to reduce noise. For spontaneous breathing experiments, tracheal pressure was measured

bioRxiv preprint doi: https://doi.org/10.64898/2026.10.22.761052; this version posted October 27, 2026. The copyright holder for this preprint (which was not certified by peer review) is the author/funder, who has granted bioRxiv a license to display the preprint in perpetuity. It is made available under a CC-BY 4.0 International license.

using an amplifier-coupled pressure transducer (Biopac) cannulated into the trachea and acquired at 1 kHz (MP150, Biopac). Pressure was detrended (Gaussian moving average, 5,000 samples), integrated over time by cumulative trapezoidal integration to obtain relative lung volume, and the integrated signal was detrended again (Savitzky–Golay, 10,000 samples). The modal tidal volume of each recording was estimated from a kernel density estimate of breath amplitudes (peak prominences), and a breath was scored if the lung-volume excursion reached ≥30% of this modal tidal volume with a minimum inter-breath interval of 0.2 s; end-inspiratory maxima and end-expiratory minima defined each breath. Respiratory rate was calculated from consecutive end-inspiratory intervals, tidal volume was taken as the end-inspiratory peak prominence of the integrated trace, heart rate was calculated from R-wave intervals of a baseline-corrected, rectified EKG, and all variables were expressed as the percent change between the 20 s preceding and the 10 s following each stimulus. For ventilated experiments, breathing was recorded and airway resistance and dynamic compliance were calculated from tracheal pressure and flow exactly as described above (see Airway resistance and compliance measurements), and, as for spontaneous breathing, responses were expressed as the percent change between the 20 s preceding and the 10 s following each stimulus.

Evans Blue vascular permeability assay and Wet-to-Dry Ratio Measurements

Mice were anesthetized with tribromoethanol (375 mg kg^{-1}, i.p.) 5 min before retro-orbital injection of Evans Blue (50 mg kg^{-1} in saline). Evans Blue was allowed to circulate for 10 min before optogenetic stimulation (10Hz for motor activation and 50Hz for sensory activation, 5-ms pulses, 10 s on/20 s off) for 10 min. Mice were then cardiac perfused with cold saline containing 2 mM EDTA for 10 min. Lung, upper gut (mm length), left liver lobe, skin (mm^2 area), and heart samples were homogenized in 500 µl formamide and incubated at 60°C for 12–16 h. Samples were centrifuged, and Evans Blue absorbance in the supernatant was measured at 620 and 700 nm. Absorbance at 700 nm was subtracted from the 620-nm measurement to correct for heme

bioRxiv preprint doi: https://doi.org/10.64898/2026.10.22.761052; this version posted October 27, 2026. The copyright holder for this preprint (which was not certified by peer review) is the author/funder, who has granted bioRxiv a license to display the preprint in perpetuity. It is made available under a CC-BY 4.0 International license.

background. Corrected absorbance values were normalized to the mean of control samples within each experimental cohort.

For wet-to-dry weight measurements, mice were anesthetized with tribromoethanol (375 mg kg^{-1}, i.p.) 15 min before they underwent the same optogenetic stimulation conditions as described above. Mice were then cardiac perfused with cold saline containing 2mM EDTA for 10 mins. The postcaval lung lobe, right atrium, gut (mm length), skin (mm^2 area), and mid liver lobe were collected and then weighed immediately. Samples were dried at 60°C for 72 h before dry weight was determined.

H&E Staining

Lung tissues were fixed, processed, and sectioned at 10-25 μm thickness. Sections were stained with hematoxylin and eosin (H&E) using standard histological methods by the MIT Koch Histology Facility. Whole-slide images were acquired using a digital slide scanner for histological assessment and image analysis.

Stereology, cuff measurements and nuclei density measurements

<u>Image acquisition and region definition.</u> H&E-stained 10-25 μm-thick lung lobe sections were imaged as whole slides and annotated in QuPath v0.7.0. For each section a reference annotation delimiting the lobe was drawn, together with exclusion annotations covering folds and section-edge artefact; the effective reference is the annotation minus exclusions. Regions were exported at 2 μm/px as co-registered RGB, reference-mask and exclusion-mask TIFFs. Sections were excluded from futher analysis if the effective reference was < 2 mm^2, if it was $<$ 10% of the median region area for its slide, if < 5 sampling frames were placed, or if V_V(area) > 0.85 (indicating fold, clot or debris).

<u>Stain deconvolution and tissue segmentation.</u> RGB images were separated into haematoxylin (H) and eosin (E) optical density by Ruifrok–Johnston color deconvolution[83] using the "H&E 2"

bioRxiv preprint doi: https://doi.org/10.64898/2026.10.22.761052; this version posted October 27, 2026. The copyright holder for this preprint (which was not certified by peer review) is the author/funder, who has granted bioRxiv a license to display the preprint in perpetuity. It is made available under a CC-BY 4.0 International license.

vectors (H = 0.490, 0.769, 0.410; E = 0.046, 0.842, 0.537), with $OD = -\log_{10}((I + 0.5)/255.5)$. Within the effective reference, pixels with OD(H) + OD(E) ≥ 0.15 were classified as tissue and the remainder as airspace. This single threshold defines every quantity below.

Nuclei counts: Nuclei were segmented in QuPath using InstanSeg extension v0.1.1 at the native 0.504 µm/px resolution, on a systematic-uniform-random sample of 300 µm frames using the same lattice and seed as the stereological sampling below. InstanSeg counts are reported per mm² of parenchyma (effective reference minus large lumens).

Stereology. Stereology metrics were calculated using custom Python scripts. Airspace components ≥ 200 µm equivalent diameter (large lumens) and dense tissue folds (100 µm tiles with mean OD ≥ 0.35 and ≥ 60% of pixels above that value) were excluded from the parenchymal compartment. Sampling used 300 µm frames on a 600 µm lattice with a seeded random offset, a 25 µm point grid, and six randomly rotated 250 µm line probes per frame. We report tissue volume fraction V_v by point counting, air–tissue surface density $S_v = 2 \cdot I/L$, interstitial thickness $\tau = 2 \cdot V_v/S_v$, and mean linear intercept $MLI = 4 \cdot (1 - V_v)/S_v$. Stain density is reported as mean OD per unit effective reference area. Local tissue density was additionally measured as the distribution of tissue fraction in 200 µm windows lying wholly inside the reference, with lumens and folds treated as excluded rather than empty pixels.

Airway/blood vessel detection and classification. To identify airways and blood vessels, candidate airspace components were first proposed permissively (60–4,000 µm equivalent diameter, inscribed radius ≥ 20 µm, wall occupancy ≥ 0.55 within 20 µm and ≥ 0.25 at 20–40 µm, evaluated only where the wall shell lies inside the reference). Candidates were then classified as 'vessel', 'airway', or 'neither' using a random forest (600 trees, balanced class weights). Twelve geometric and stain features were used, including rim OD expressed as a ratio to each section's own median tissue OD so that staining batch cannot influence selection. The model was trained on 1172 blind-annotated objects from 187 sections and assessed by 5-fold

bioRxiv preprint doi: https://doi.org/10.64898/2026.10.22.761052; this version posted October 27, 2026. The copyright holder for this preprint (which was not certified by peer review) is the author/funder, who has granted bioRxiv a license to display the preprint in perpetuity. It is made available under a CC-BY 4.0 International license.

cross-validation grouped by section, giving an overall accuracy of 0.84 (precision and recall 0.62 and 0.66 for airways, 0.66 and 0.55 for vessels). Lumens were analysed at $P(\text{vessel}) + P(\text{airway}) \geq 0.60$; vessels were defined as $P(\text{vessel}) > 0.5$; airways were defined permissively as $P(\text{airway}) > P(\text{vessel})$. Detection was verified against a per-region overlay generated from the same masks used for measurement. The trained classifier, its SHA-256 digest and the blinded label set are archived with the code.

Cuff thickness calculations. Collar thickness was measured as the Euclidean distance from each point on the one-pixel shell outside a detected lumen to the nearest airspace that is not itself a detected lumen, sampled at full resolution and suppressed within 10 μm of the reference boundary. Per lumen we report the median of this distribution. Analyses are stratified by lumen caliber using both area-equivalent and maximum-inscribed diameter, since the former overstates the bore of elongated and branched profiles. For caliber sweeps, a single omnibus test per lumen class was obtained by exhaustive enumeration of all 9,240 within-block reassignments of the treatment label, giving an exact P value, with a family-wise-corrected $\max|t|$ statistic identifying the caliber band contributing most. Negative results are reported as effect sizes with 95% confidence intervals rather than as $P > 0.05$.

Statistics: N refers to animals throughout, and no test treats sections, lumens or pixels as independent. Per-section values were aggregated to the animal as region-area–weighted means; collar thickness was aggregated as the median of per-lumen medians. Group comparisons used two-sided Welch t-tests on per-animal values, and every contrast is additionally reported from an OLS model including tissue volume fraction as a covariate, to account for differences in inflation states. Where both arms of a contrast were present in more than one staining round or slide block, that factor was included as a fixed effect and the blocked estimate is the one reported; contrasts in which a group is absent from some strata are flagged as confounded rather than pooled. Timepoints were never concatenated.

bioRxiv preprint doi: https://doi.org/10.64898/2026.10.22.761052; this version posted October 27, 2026. The copyright holder for this preprint (which was not certified by peer review) is the author/funder, who has granted bioRxiv a license to display the preprint in perpetuity. It is made available under a CC-BY 4.0 International license.

Software versions: Analysis used Python 3.10.12 with NumPy 2.2.6, pandas 2.3.3, SciPy 1.15.3, scikit-image 0.25.2, scikit-learn 1.7.2, statsmodels 0.15.0 and Matplotlib 3.10.9;

UEA-1+ area/ airspace measurements:

UEA-1 staining was quantified using custom MATLAB scripts. Images were analyzed in a fixed, randomly permuted order (fixed seed, saved to disk) to minimize bias. For each field, the full airway (lumen, wall, and epithelium) was manually outlined on the structural reference channel to generate a binary airway mask; masks were saved and all measurements were computed from the saved masks and raw images. Mucus-positive pixels were defined by a fixed intensity threshold (>0.10 of the normalized intensity range) applied to the UEA-1 channel, and mucus density was calculated as UEA-1–positive pixels within the airway mask per 1,000 airway pixels. A 300-dpi quality-control overlay of the airway boundary and UEA-1 signal was generated and reviewed for every image.

CD45 area/ DAPI area measurements:

CD45$^+$ immune infiltration was quantified in whole lung lobe sections (5× confocal, channel 1 = DAPI, channel 2 = CD45) using a custom MATLAB script. Images were analyzed blinded, with coded identifiers assigned in randomized order and the identifier-to-filename key withheld until all images were complete. Folds and artifactual regions were manually outlined and excluded, and lung tissue was defined as DAPI$^+$ pixels (intensity > 0.015) outside excluded regions. CD45$^+$ pixels were identified with a local adaptive threshold equal to the Gaussian-smoothed local background (σ = 10 px) plus 0.5 × the local Gaussian-smoothed absolute deviation from that background, subject to a hard minimum of 0.05 normalized intensity; objects < 3 px were discarded. CD45$^+$ burden was expressed as CD45$^+$ pixel area divided by DAPI$^+$ lung area, with CD45$^+$ object count per lung area as a secondary measure. Every image was reviewed against

bioRxiv preprint doi: https://doi.org/10.64898/2026.10.22.761052; this version posted October 27, 2026. The copyright holder for this preprint (which was not certified by peer review) is the author/funder, who has granted bioRxiv a license to display the preprint in perpetuity. It is made available under a CC-BY 4.0 International license.

a six-panel segmentation QC montage, and any threshold adjustment was made blinded and logged.

Color deconvolution of Evans blue and blood signal: RGB images of whole lung lobes were separated into component channels in Fiji using the Colour Deconvolution plugin, which implements the stain-unmixing method of Ruifrok and Johnston (ref). Because Evans blue and hemoglobin are not standard histological stains, a custom three-component stain vector matrix was defined empirically: regions containing predominantly Evans blue, predominantly blood, or unstained background tissue were sampled from control images, and their mean RGB values were entered as the three stain vectors (exact R, G, B coordinates for each component are given in table S2). The identical vector matrix was applied to every image across all experimental groups. Deconvolution produced separate 8-bit Evans blue and blood channels, with the third component capturing residual background. All lobes were imaged under identical illumination, exposure, and white-balance settings.

In situ Hybridization

Fluorescence in situ hybridization was performed using the HCR v3.0 protocol (Molecular Instruments) to detect *Cysltr2* and *ChR2* transcripts. Tissue sections (14um) were fixed, permeabilized, and incubated with target-specific HCR probe sets against *Cysltr2* and *ChR2*, followed by hybridization with fluorescently labeled HCR hairpins according to the manufacturer's protocol. Sections were washed and mounted for fluorescence imaging. Probe sets were obtained from Molecular Instruments.

Calcium Imaging of dissociated vagal sensory neurons

Nodose/jugular ganglia were resected into ice-cold DMEM, cleaned of connective tissue and nerve rootlets, and pooled on ice; ganglia from 1 mouse yielded sufficient neurons for several rounds of imaging in a single day. Ganglia were settled by gravity, the DMEM was aspirated,

bioRxiv preprint doi: https://doi.org/10.64898/2026.10.22.761052; this version posted October 27, 2026. The copyright holder for this preprint (which was not certified by peer review) is the author/funder, who has granted bioRxiv a license to display the preprint in perpetuity. It is made available under a CC-BY 4.0 International license.

and ganglia were digested in DMEM containing 0.11 mg/mL Liberase TH (collagenase I/II–thermolysin blend; Roche 5401135001) and 0.004% DNase I (Worthington LS002007 or recombinant DNase I, Roche 04716728001) for 45 min at 37 °C with rotation, then pelleted (300 × g, 5 min, 4 °C, swinging-bucket rotor).

Ganglia were resuspended in ice-cold 1× low-ovomucoid inhibitor solution (0.15% BSA [Sigma-Aldrich A8806], 0.15% ovomucoid trypsin inhibitor [Worthington LS003086], and 0.004% DNase I in DMEM, pH 7.4, 0.22-μm filtered) and mechanically dissociated by ~15–20 gentle passages with a P200 pipette held flat against the tube bottom, avoiding bubbles, until the medium became cloudy. The suspension was diluted in the same solution, filtered through a pre-wetted 70-μm strainer, pelleted (300 × g, 5 min, 4 °C), washed once more in low-ovomucoid solution, and re-pelleted.

Neurons were resuspended at high density in low-ovomucoid solution and spot-seeded as small droplets onto the center of dry, pre-coated poly-D-lysine/laminin 12-mm cover glasses (Neuvitro GG-12-laminin) to concentrate cells within a single field of view. Cells attached for 10 min at 37 °C in a humidified incubator, after which pre-warmed medium was layered on top to prevent desiccation.

For imaging, cover glasses were mounted in a perfusion chamber and superfused continuously with room temperature HBSS containing Ca^{2+} and Mg^{2+}, which is required for robust agonist-evoked responses. Solutions were delivered by gravity flow at a constant ~7 mL/min with gravity evacuation, with flow rate consistent across all solutions. Epifluorescent images were acquired at 8 Hz (125 ms per frame) through a 5× air objective, which captured the entire seeded field in a single frame; tdTomato was imaged in a separate channel to assign *Cysltr2*-lineage identity to each region of interest. All stimuli were prepared in HBSS with Ca^{2+} and Mg^{2+} and applied sequentially by switching the perfusion line, with HBSS washout between applications: histamine (Millipore Sigma, 1 μM), LTC_4 and LTD_4 cocktail (cysteinyl leukotrienes; Cayman Chemical Company, 100nM), allyl isothiocyanate (AITC; Millipore Sigma, 100 μM), bradykinin

bioRxiv preprint doi: https://doi.org/10.64898/2026.10.22.761052; this version posted October 27, 2026. The copyright holder for this preprint (which was not certified by peer review) is the author/funder, who has granted bioRxiv a license to display the preprint in perpetuity. It is made available under a CC-BY 4.0 International license.

(Millipore Sigma, 1 nM), Yoda1 (Tocris, 10 μM), serotonin (Millipore Sigma, 10 μM), and capsaicin (Millipore Sigma, 2 μM). KCl (Sigma Aldrich, 50 mM) was applied last in every experiment as a positive control to identify all viable neurons.

Sympathetic ablation

Mice received 6-hydroxydopamine (6-OHDA; Cayman Chemical Company, 100 mg kg^{-1} on day −6 and 250 mg kg^{-1} on day −4) in 0.1% ascorbic acid (Sigma Aldrich) in saline via intraperitoneal injection. Evans Blue vascular permeability was assessed on day 0 as described above.

bioRxiv preprint doi: https://doi.org/10.64898/2026.10.22.761052; this version posted October 27, 2026. The copyright holder for this preprint (which was not certified by peer review) is the author/funder, who has granted bioRxiv a license to display the preprint in perpetuity. It is made available under a CC-BY 4.0 International license.

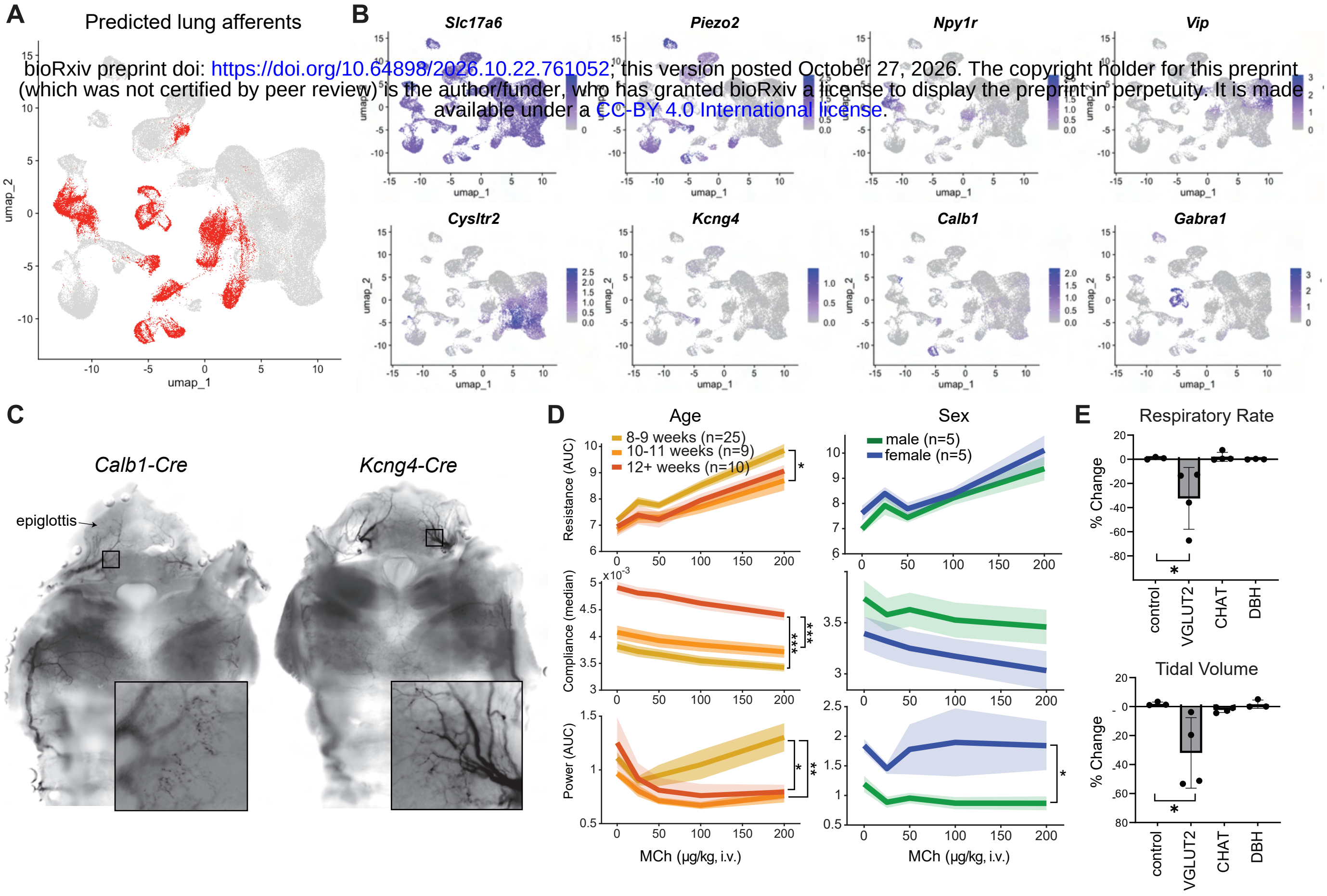

Figure S1

bioRxiv preprint doi: https://doi.org/10.64898/2026.10.22.761052; this version posted ... The copyright holder for this preprint (which was not certified by peer review) is the author/funder, who has granted bioRxiv a license to display the preprint in perpetuity. It is made available under a CC-BY 4.0 International license.

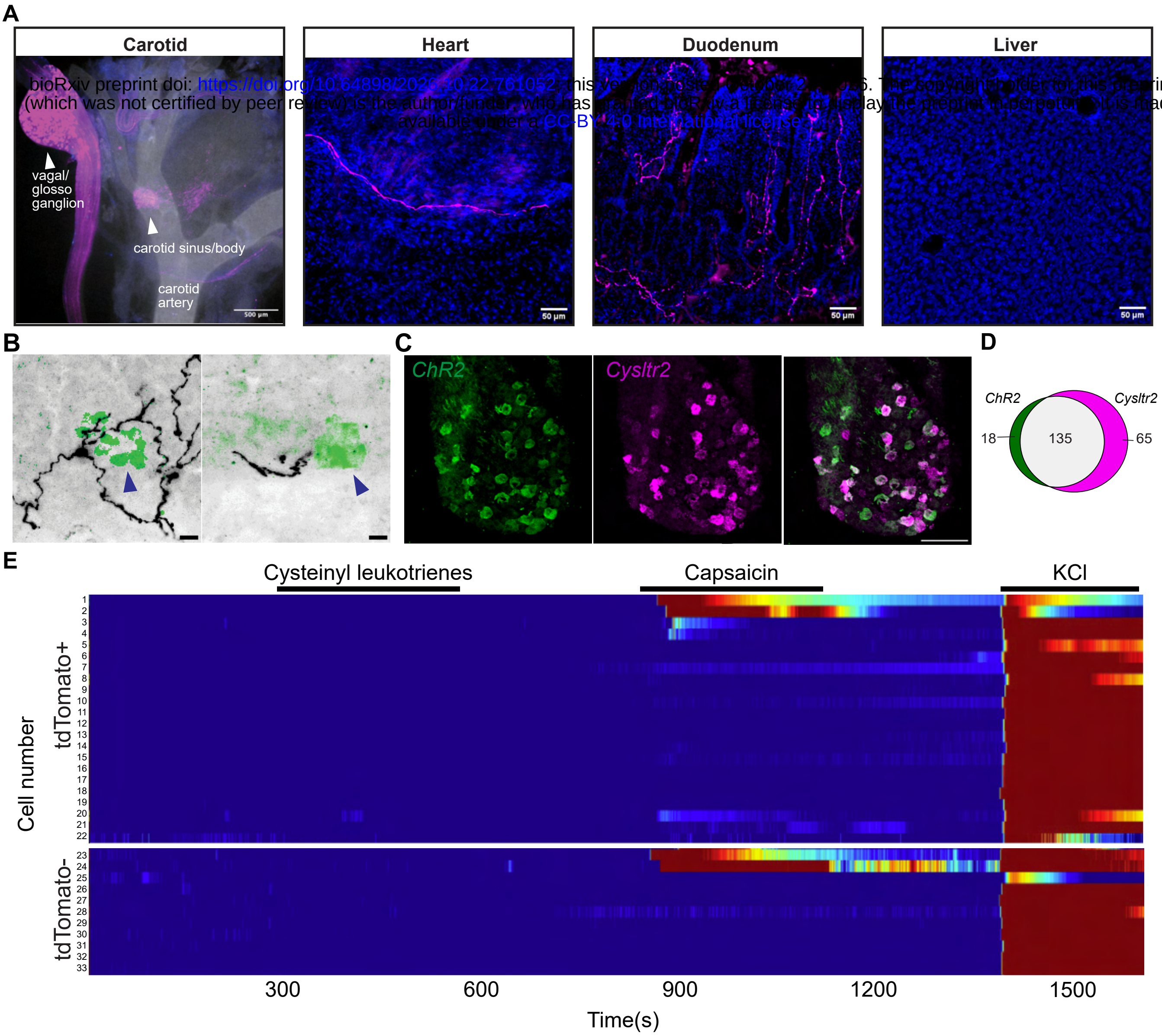

Figure S2

bioRxiv preprint doi: https://doi.org/10.64898/2026.10.22.761052; this version posted October 27, 2026. The copyright holder for this preprint (which was not certified by peer review) is the author/funder, who has granted bioRxiv a license to display the preprint in perpetuity. It is made available under a CC-BY 4.0 International license.

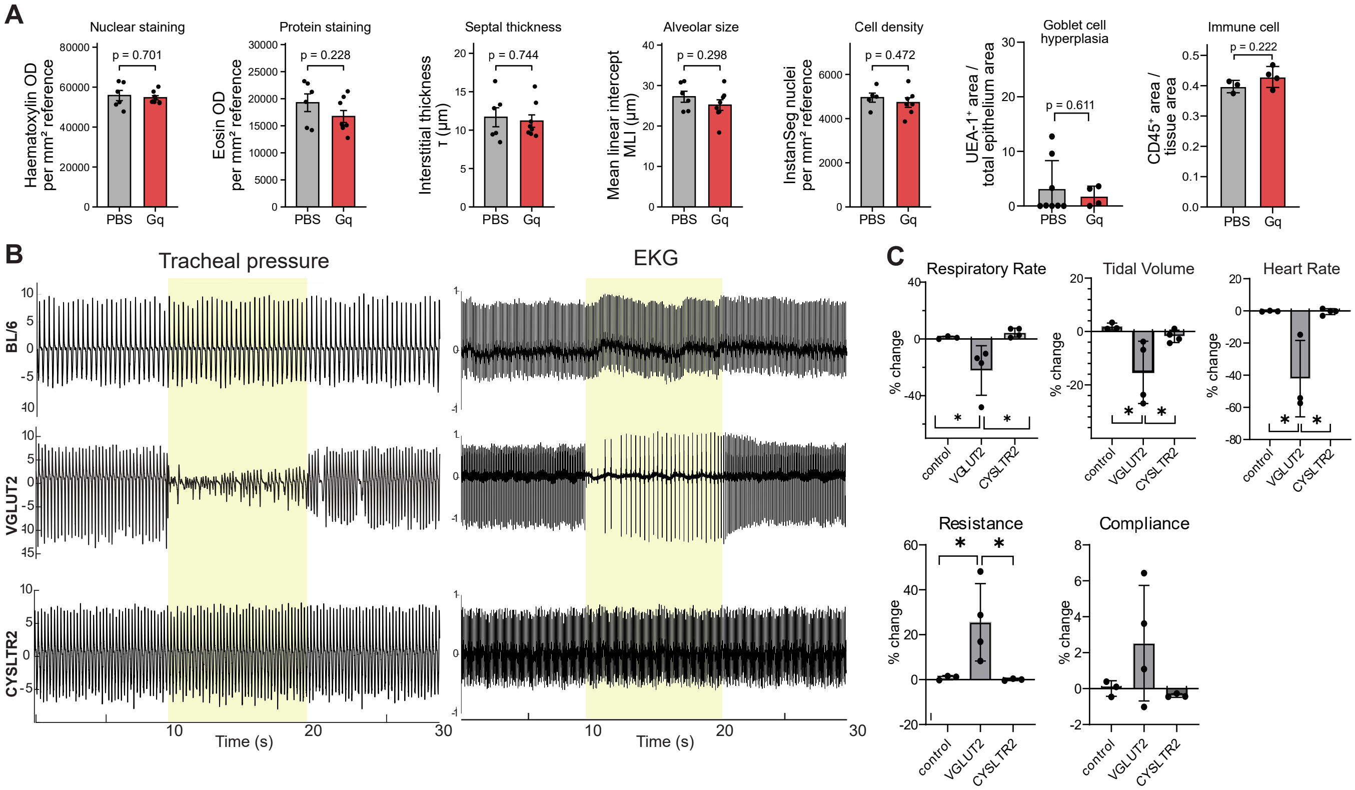

Figure S3

bioRxiv preprint doi: https://doi.org/10.64898/2026.10.22.761052; this version posted October 27, 2026. The copyright holder for this preprint (which was not certified by peer review) is the author/funder, who has granted bioRxiv a license to display the preprint in perpetuity. It is made available under a CC-BY 4.0 International license.

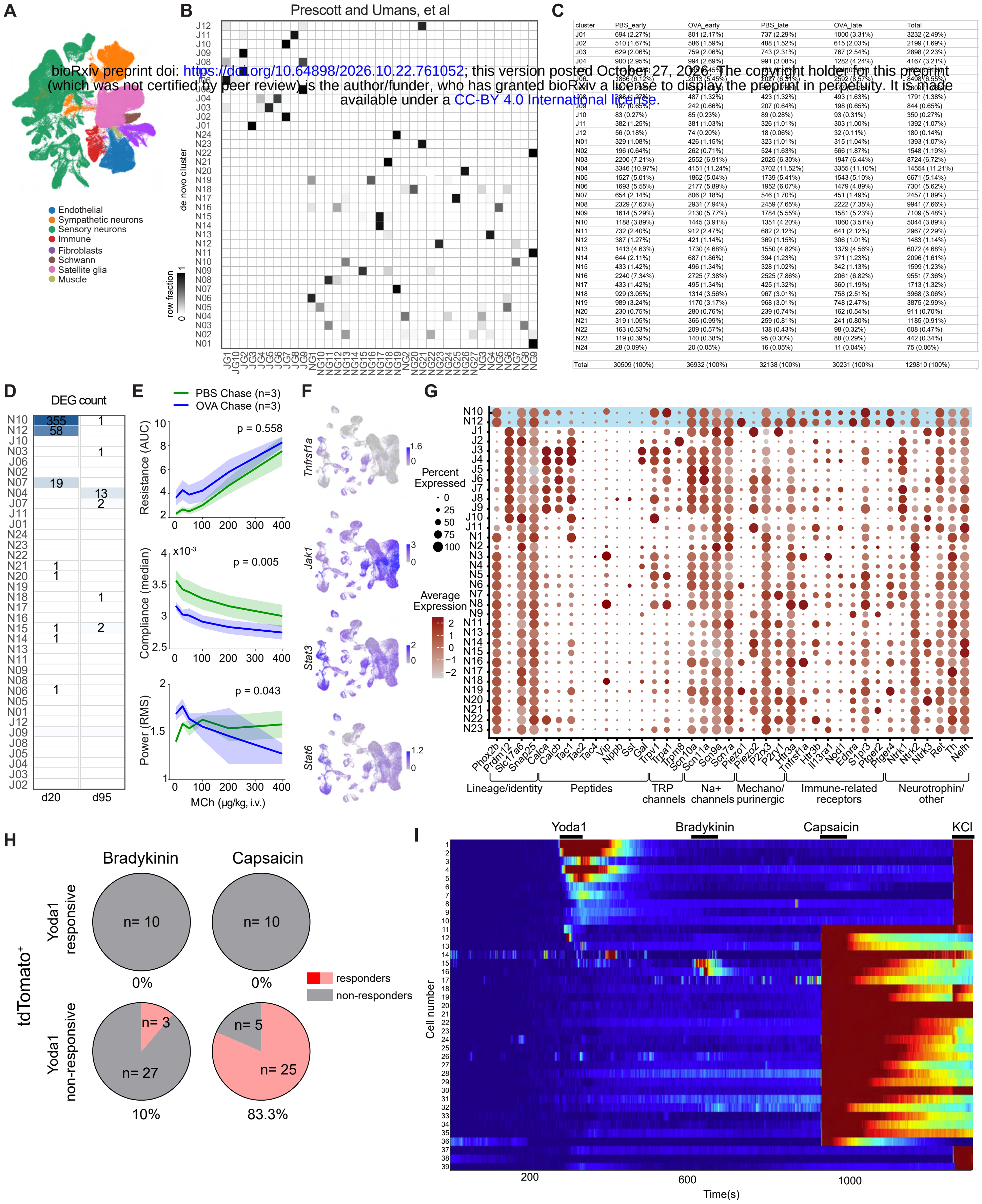

cluster	PBS_early	OVA_early	PBS_late	OVA_late	Total
J01	694 (2.27%)	801 (2.17%)	737 (2.29%)	1000 (3.31%)	3232 (2.49%)
J02	510 (1.67%)	586 (1.59%)	488 (1.52%)	615 (2.03%)	2199 (1.69%)
J03	629 (2.06%)	759 (2.06%)	743 (2.31%)	767 (2.54%)	2898 (2.23%)
J04	900 (2.95%)	994 (2.69%)	991 (3.08%)	1282 (4.24%)	4167 (3.21%)
J05	[illegible]	[illegible]	[illegible]	[illegible]	[illegible]
J06	1866 (6.12%)	2013 (5.45%)	2027 (6.31%)	2592 (8.57%)	8498 (6.55%)
J07	527 (1.73%)	[illegible]	[illegible]	[illegible]	[illegible]
J08	388 (1.27%)	487 (1.32%)	423 (1.32%)	493 (1.63%)	1791 (1.38%)
J09	197 (0.65%)	242 (0.66%)	207 (0.64%)	198 (0.65%)	844 (0.65%)
J10	83 (0.27%)	85 (0.23%)	89 (0.28%)	93 (0.31%)	350 (0.27%)
J11	382 (1.25%)	381 (1.03%)	326 (1.01%)	303 (1.00%)	1392 (1.07%)
J12	56 (0.18%)	74 (0.20%)	18 (0.06%)	32 (0.11%)	180 (0.14%)
N01	329 (1.08%)	426 (1.15%)	323 (1.01%)	315 (1.04%)	1393 (1.07%)
N02	196 (0.64%)	262 (0.71%)	524 (1.63%)	566 (1.87%)	1548 (1.19%)
N03	2200 (7.21%)	2552 (6.91%)	2025 (6.30%)	1947 (6.44%)	8724 (6.72%)
N04	3346 (10.97%)	4151 (11.24%)	3702 (11.52%)	3355 (11.10%)	14554 (11.21%)
N05	1527 (5.01%)	1862 (5.04%)	1739 (5.41%)	1543 (5.10%)	6671 (5.14%)
N06	1693 (5.55%)	2177 (5.89%)	1952 (6.07%)	1479 (4.89%)	7301 (5.62%)
N07	654 (2.14%)	806 (2.18%)	546 (1.70%)	451 (1.49%)	2457 (1.89%)
N08	2329 (7.63%)	2931 (7.94%)	2459 (7.65%)	2222 (7.35%)	9941 (7.66%)
N09	1614 (5.29%)	2130 (5.77%)	1784 (5.55%)	1581 (5.23%)	7109 (5.48%)
N10	1188 (3.89%)	1445 (3.91%)	1351 (4.20%)	1060 (3.51%)	5044 (3.89%)
N11	732 (2.40%)	912 (2.47%)	682 (2.12%)	641 (2.12%)	2967 (2.29%)
N12	387 (1.27%)	421 (1.14%)	369 (1.15%)	306 (1.01%)	1483 (1.14%)
N13	1413 (4.63%)	1730 (4.68%)	1550 (4.82%)	1379 (4.56%)	6072 (4.68%)
N14	644 (2.11%)	687 (1.86%)	394 (1.23%)	371 (1.23%)	2096 (1.61%)
N15	433 (1.42%)	496 (1.34%)	328 (1.02%)	342 (1.13%)	1599 (1.23%)
N16	2240 (7.34%)	2725 (7.38%)	2525 (7.86%)	2061 (6.82%)	9551 (7.36%)
N17	433 (1.42%)	495 (1.34%)	425 (1.32%)	360 (1.19%)	1713 (1.32%)
N18	929 (3.05%)	1314 (3.56%)	967 (3.01%)	758 (2.51%)	3968 (3.06%)
N19	989 (3.24%)	1170 (3.17%)	968 (3.01%)	748 (2.47%)	3875 (2.99%)
N20	230 (0.75%)	280 (0.76%)	239 (0.74%)	162 (0.54%)	911 (0.70%)
N21	319 (1.05%)	366 (0.99%)	259 (0.81%)	241 (0.80%)	1185 (0.91%)
N22	163 (0.53%)	209 (0.57%)	138 (0.43%)	98 (0.32%)	608 (0.47%)
N23	119 (0.39%)	140 (0.38%)	95 (0.30%)	88 (0.29%)	442 (0.34%)
N24	28 (0.09%)	20 (0.05%)	16 (0.05%)	11 (0.04%)	75 (0.06%)
Total	30509 (100%)	36932 (100%)	32138 (100%)	30231 (100%)	129810 (100%)

Figure S4

bioRxiv preprint doi: https://doi.org/10.64898/2026.10.22.761052; this version posted October 27, 2026. The copyright holder for this preprint (which was not certified by peer review) is the author/funder, who has granted bioRxiv a license to display the preprint in perpetuity. It is made available under a CC-BY 4.0 International license.

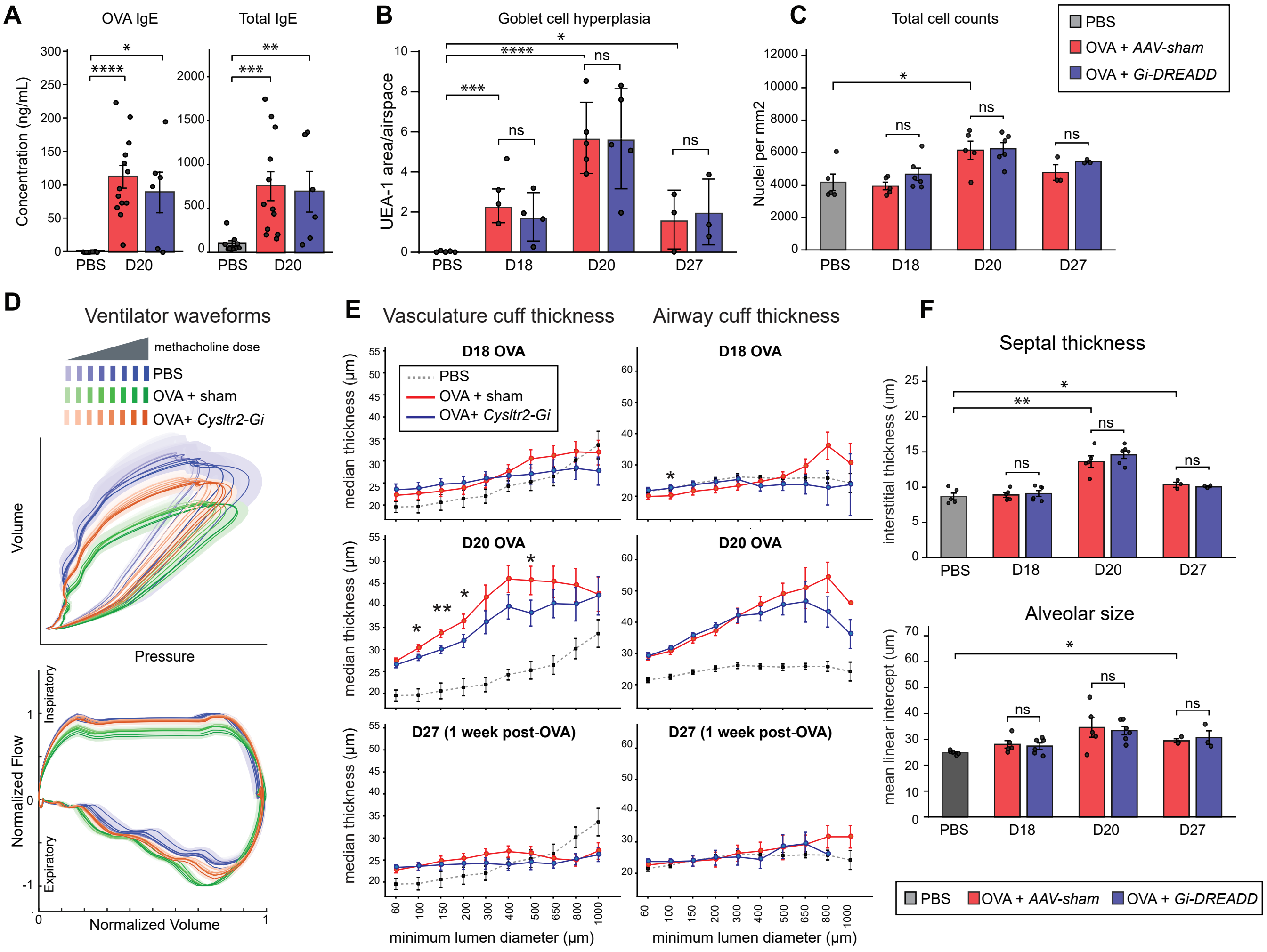

Figure S5

bioRxiv preprint doi: https://doi.org/10.64898/2026.10.22.761052; this version posted October 27, 2026. The copyright holder for this preprint (which was not certified by peer review) is the author/funder, who has granted bioRxiv a license to display the preprint in perpetuity. It is made available under a CC-BY 4.0 International license.

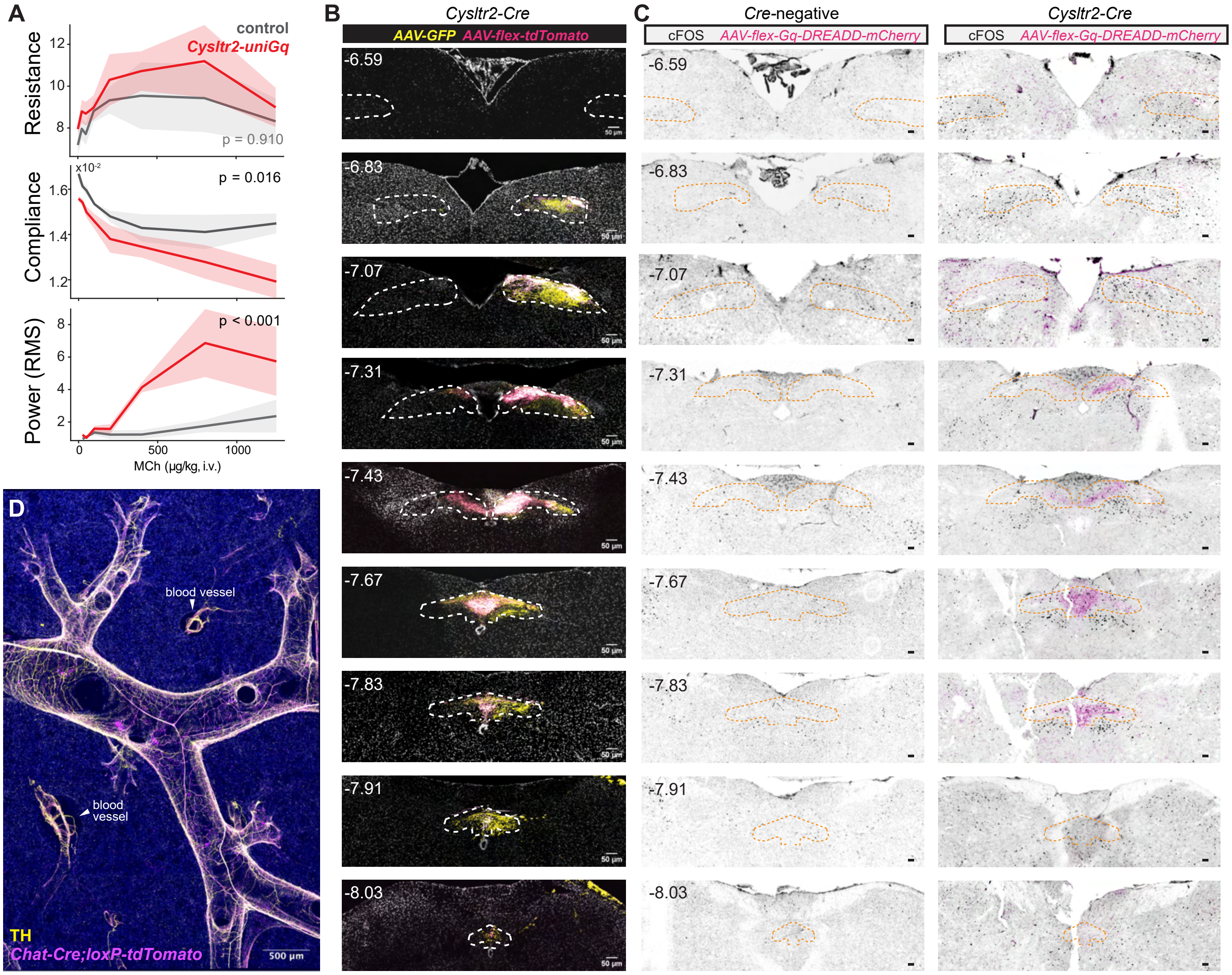

Figure S6

bioRxiv preprint doi: https://doi.org/10.64898/2026.10.22.761052; this version posted October 27, 2026. The copyright holder for this preprint (which was not certified by peer review) is the author/funder, who has granted bioRxiv a license to display the preprint in perpetuity. It is made available under a CC-BY 4.0 International license.

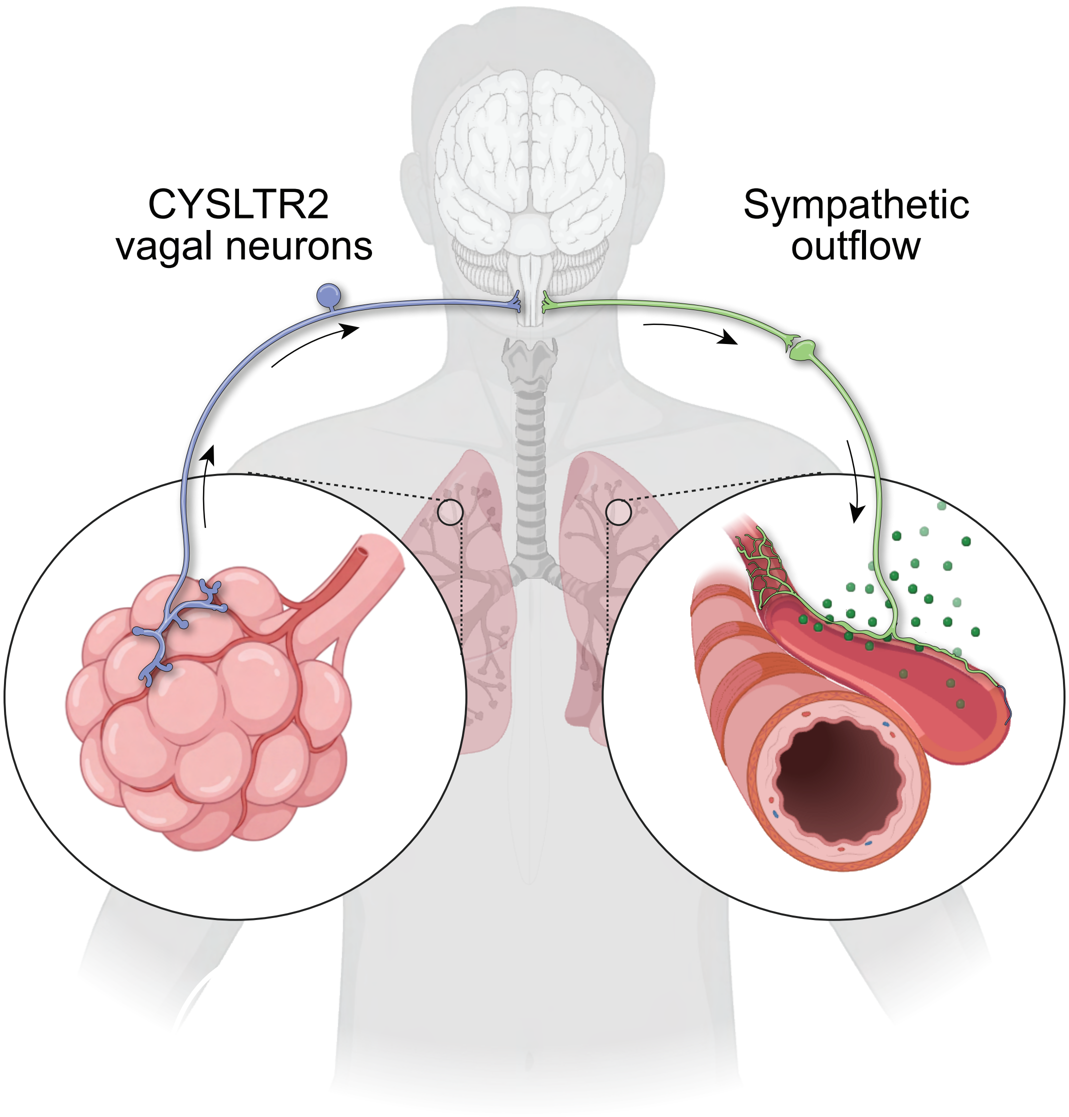

Figure S7
